# Supplementary material for: Longitudinal body mass index and cancer risk: a cohort study of 2.6 million Catalan adults
Source: Nat Commun. 2023 Jun 30;14:3816. doi: 10.1038/s41467-023-39282-y (PMC10313757; doi:10.1038/s41467-023-39282-y)
Supplement: Supplementary file 1 — Supplementary information [file 41467_2023_39282_MOESM1_ESM.pdf]

Supplementary Material

Longitudinal body mass index-derived exposures during early adulthood and risk of 26 types of cancer: a cohort study of 2.6 million adults in Catalonia, Spain

Martina Recalde[1,2,3†], Andrea Pistillo[2†], Veronica Davila-Batista[1,4*], Michael Leitzmann[5], Isabelle Romieu[6], Vivian Viallon[1], Heinz Freisling[1††*], Talita Duarte-Salles[2††*]

†These authors contributed equally
††These authors jointly supervised this work
*Corresponding authors

1. International Agency for Research on Cancer (IARC-WHO), 25 avenue Tony Garnier, CS 90627, 69366 Lyon Cedex 07, France
2. Fundació Institut Universitari per a la recerca a l'Atenció Primària de Salut Jordi Gol i Gurina (IDIAPJGol), Barcelona, Spain
3. Universitat Autònoma de Barcelona, Spain
4. Spanish Consortium for Research on Epidemiology and Public Health (CIBERESP), Instituto de Salud Carlos III, 28029, Madrid, Spain
5. Department of Epidemiology and Preventive Medicine, University of Regensburg, Regensburg, Germany
6. Center for Research on Population Health, National Institute of Public Health, Mexico City, Mexico

Corresponding authors:
Talita Duarte-Salles
tduarte@idiapjgol.org
Fundació Institut Universitari per a la recerca a l'Atenció Primària de Salut Jordi Gol i Gurina (IDIAPJGol)
Gran Via Corts Catalanes, 587 àtic
08007 Barcelona, Spain

Heinz Freisling
FreislingH@iarc.fr
International Agency for Research on Cancer (IARC-WHO)
25 avenue Tony Garnier, CS 90627,
69366 LYON CEDEX 07, France

Veronica Davila-Batista
vdavbat@gobiernodecanarias.org
Endocrinology Department, Complejo Hospitalario Universitario Insular Materno Infantil, Las Palmas de GC, Canary Health Service
Av. Marítima s/n
35016 - Las Palmas de Gran Canaria, Spain

Figure S1. Flowchart with the inclusion and exclusion criteria of the study population for the time-to-event analysis 3

Table S1. Baseline characteristics of the study population with and without a BMI assessment 4

Table S2. Hazard ratios of 26 cancer types related to one standard deviation increment of BMI at index date and other longitudinal BMI-derived exposures, with 95% CIs 6

Table S3. Standard deviation of BMI at index date and other longitudinal BMI-derived exposures, by subgroups of participants 8

Figure S2. Hazard ratios of 26 cancer types related to BMI at index, with 95% CIs, allowing for non-linearity 9

Figure S3. Forest plot of hazard ratios of 26 cancer types related to one standard deviation increment of BMI at index date and other longitudinal BMI-derived exposures, minimally adjusted models, with 95% CIs 11

Appendix 1. Description of the secondary and sensitivity analyses of this study 13

Figure S4. Hazard ratios of 26 cancer types related to one standard deviation increment of BMI at index date and other longitudinal BMI-derived exposures stratified by age and sex, with 95% CIs 14

Figure S5. Hazard ratios of 26 cancer types related to one standard deviation increment of BMI at index date and other longitudinal BMI-derived exposures with mutual adjustment of duration of BMI ≥25 (≥30) kg/m^2 and age of onset of BMI ≥25 (≥30) kg/m^2, respectively, with 95% CIs 17

Figure S6. Pearson's correlation matrix between exposures 19

Figure S7. Hazard ratios of 26 cancer types related to one standard deviation increment of BMI at index date and other longitudinal BMI-derived exposures restricted to never smokers, with 95% CIs 20

Figure S8. Changes in risk discrimination for the risk of 26 cancer types after addition of longitudinal BMI-derived exposures to the fully-adjusted model including BMI at index date as the main exposure 22

Figure S9. Hazard ratios of 26 cancer types related to one standard deviation increment of BMI at index date and other longitudinal BMI-derived exposures restricted to BMIs ≥25 and <30 kg/m^2, with 95% CIs 24

Figure S10. Hazard ratios of 26 cancer types related to one standard deviation increment of BMI at index date and other longitudinal BMI-derived exposures in sensitivity analyses, with 95% CIs 26

Figure S11. Hazard ratios of 26 cancer types related to one standard deviation increment of BMI at index date and other longitudinal BMI-derived exposures in sensitivity analyses, with Bonferroni correction 29

Figure S12. Summary of the findings of this study and the Viewpoint of the IARC Working Group on Body Fatness and Cancer. 31

Table S4. Diagnostic codes for the definition of cancer cases 33

Appendix 2. Description of the multiple imputations' methodology for missing data on body mass index and the covariates of interest 35

Figure S13. Timeline of this study: illustration with the example of two study participants 38

Figure S14. Graphical representation for the obtention of longitudinal exposures from BMI trajectories: illustration with the example of an imputed trajectory from a study participant 39

Figure S15. Directed Acyclic Graph for the possible causal effect of body fatness on cancer used to adjust the Cox proportional hazard models 40

Table S5. N of cases for those who ever lived with a BMI ≥25 and/or ≥30 kg/m^2 throughout the different analyses performed in this study 41

Table S6. Baseline characteristics of the study population, overall and by having ever had a body mass index ≥25 or ≥30 kg/m^2, after multiple imputations 43

Appendix 3. STROBE Statement checklist 45

Figure S1. Flowchart with the inclusion and exclusion criteria of the study population for the time-to-event analysis

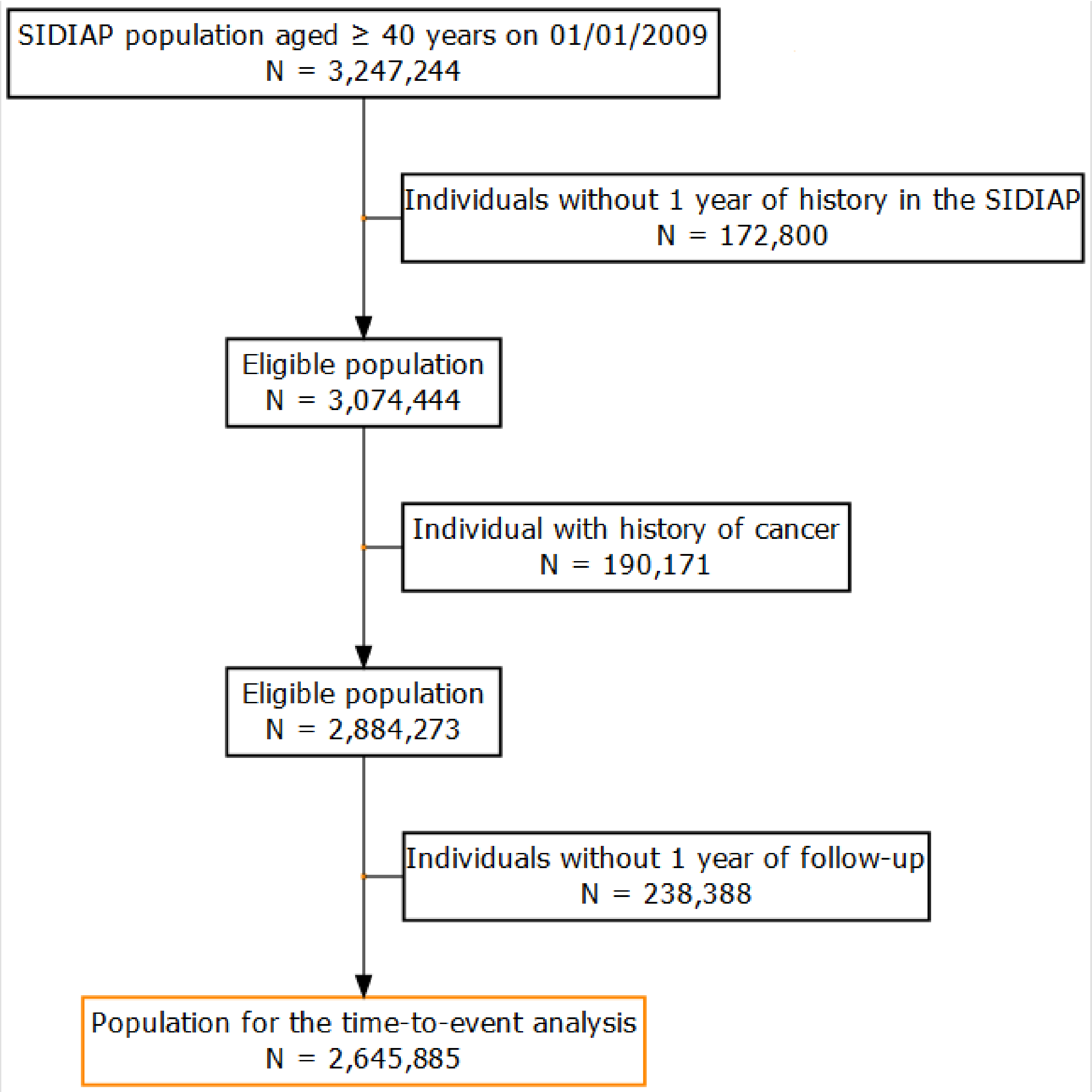

Notes: History of cancer considers any type of cancer (C00-C97) except C44 (other and unspecified malignant neoplasm of skin). Causes of end-of-follow-up include transferral out of SIDIAP, cancer diagnosis, death, or end-of-study period. Individuals with less thant 1 year of follow-up were excluded because the follow-up of the participants started 1 year after study entry to avoid potential reverse causality (eg, BMI affected by undiagnosed cancer).

Abbreviations: SIDIAP: Information System for Research in Primary Care.

Table S1. Baseline characteristics of the study population with and without a BMI assessment

	Main Dataset N (%)	Individuals with at least one BMI assessment N (%)[1]	Individuals without BMI assessment N (%)[1]
	2,645,885 (100)	2,081,840 (78.7)	564,045 (21.3)
**Follow-up time in years, median (IQR)**	9.0 (7.7, 9.0)	9.0 (9.0, 9.0)	9.0 (4.4, 9.0)
**Duration of BMI ≥25 kg/m^2 in years, median (IQR)[2]**	12.0 (0.0, 23.0)	12.0 (0.0, 23.0)	11.0 (0.0, 23.0)
**Duration of BMI ≥30 kg/m^2 in years, median (IQR)[2]**	0.0 (0.0, 4.0)	0.0 (0.0, 4.0)	0.0 (0.0, 3.0)
**Cumulative exposure to BMI ≥25 kg/m^2 in cumulative overweight-years, median (IQR)[2,3]**	16.4 (0.0, 73.7)	16.8 (0.0, 74.4)	15.1 (0.0, 71.5)
**Cumulative exposure to BMI ≥30 kg/m^2 in cumulative obese-years, median (IQR)[2,3]**	0.0 (0.0, 2.2)	0.0 (0.0, 2.4)	0.0 (0.0, 1.7)
**Age of onset of BMI ≥25 kg/m^2 in years, median (IQR)[2,4]**	20.0 (18.0, 29.0)	20.0 (18.0, 29.0)	20.0 (18.0, 28.0)
**Age of onset of BMI ≥30 kg/m^2 in years, median (IQR)[2,4]**	29.0 (21.0, 35.0)	28.0 (20.0, 35.0)	29.0 (23.0, 35.0)
**BMI at index date in kg/m^2, median (IQR)[2,5]**	27.6 (24.2, 31.1)	27.7 (24.4, 31.2)	27.2 (23.6, 30.7)
**Age in years, median (IQR)**	56.0 (47.0, 68.0)	58.0 (48.0, 69.0)	51.0 (44.0, 65.0)
**Male sex, n (%)**	1,241,523 (46.9)	960,085 (46.1)	281,438 (49.9)
**Nationality**			
Spanish	2,495,536 (94.3)	1,989,049 (95.5)	506,487 (89.8)
Global North	51,320 (1.9)	30,266 (1.5)	21,054 (3.7)
Global South	99,029 (3.7)	62,525 (3.0)	36,504 (6.5)
**MEDEA deprivation index, n (%)[2]**			
Quintile 1 (least deprived)	472,049 (17.8)	343,753 (16.5)	128,296 (22.7)
Quintile 2	429,823 (16.2)	334,335 (16.1)	95,488 (16.9)
Quintile 3	416,465 (15.7)	332,173 (16.0)	84,292 (14.9)
Quintile 4	401,681 (15.2)	324,939 (15.6)	76,742 (13.6)
Quintile 5 (most deprived)	361,665 (13.7)	292,949 (14.1)	68,717 (12.2)
Rural	564,201 (21.3)	453,691 (21.8)	110,511 (19.6)
**Smoking status, n (%)[2]**			
Never smoker	1,663,154 (62.9)	1,326,374 (63.7)	336,780 (59.7)
Former smoker	390,711 (14.8)	321,988 (15.5)	68,723 (12.2)

Current smoker	592,020 (22.4)	433,477 (20.8)	158,542 (28.1)
**Alcohol intake, n (%)²**			
No risk	1,663,281 (62.9)	1,307,940 (62.8)	355,341 (63.0)
Low risk	894,238 (33.8)	704,688 (33.8)	189,550 (33.6)
High risk	88,366 (3.3)	69,212 (3.3)	19,154 (3.4)
**Charlson comorbidity index, n (%)**			
0	1,250,781 (47.3)	841,352 (40.4)	409,429 (72.6)
1	892,103 (33.7)	796,407 (38.3)	95,696 (17.0)
2	357,217 (13.5)	316,445 (15.2)	40,772 (7.2)
≥ 3	145,784 (5.5)	127,636 (6.1)	18,148 (3.2)
**Cause of exit from the study, n (%)**			
End of study	1,865,496 (70.5)	1,564,616 (75.2)	300,880 (53.3)
Transferred out of the SIDIAP	291,641 (11.0)	133,364 (6.4)	158,277 (28.1)
Death	250,914 (9.5)	176,764 (8.5)	74,150 (13.1)
Any cancer	237,834 (9.0)	207,096 (9.9)	30,738 (5.4)
**Cancer outcomes, n (%)**	225,396 (8.5)	196,505 (9.4)	28,891 (5.1)

Notes: 1) This categorization was done in the 5 datasets obtained after performing the multiple imputations. For visualization purposes and in order for the categorical variables to add up to 2,645,885 we divided the n for the categorical variables by 5. 2) The exposures of interest, the MEDEA deprivation index, smoking status, and alcohol intake were calculated using the multiple imputation approach, with 5 data sets created. For visualization purposes, we divided the n for the categorical variables by 5. 3) This indicator was calculated by adding the difference between the BMI measurements that were ≥25 (≥30, for obesity) kg/m^2 and 24.9 (29.9) kg/m^2 for every year lived with a BMI ≥25 and ≥30, respectively. 4) Age of onset of a BMI ≥25 (and ≥30) kg/m^2 is only available for individuals who ever had a BMI ≥25 (≥30) kg/m^2. 5) BMI assessment at the start of the time-to-event analysis (baseline BMI). 6) Any cancer does not include non-melanoma skin cancer.

Abbreviations: BMI: Body Mass Index; IQR: Interquartile range; MEDEA: “Mortalidad en áreas pequeñas Españolas y Desigualdades Socioeconómicas y Ambientales”; SIDIAP: Information System for Research in Primary Care.

Table S2. Hazard ratios of 26 cancer types related to one standard deviation increment of BMI at index date and other longitudinal BMI-derived exposures, with 95% CIs

	Hazard Ratio (95% CI) for each exposure						
**Cancer type**	**BMI at index**	**Duration of BMI ≥25 kg/m²**	**Duration of BMI ≥30 kg/m²**	**Cumulative exposure to BMI ≥25 kg/m²**	**Cumulative exposure to BMI ≥30 kg/m²**	**Age onset of BMI ≥25 kg/m²**	**Age onset of BMI ≥30 kg/m²**
**Corpus Uteri**	1.55 (1.51-1.58)	1.46 (1.42-1.51)	1.42 (1.38-1.46)	1.42 (1.39-1.45)	1.29 (1.27-1.31)	1.35 (1.29-1.41)	1.40 (1.32-1.49)
**Kidney**	1.15 (1.12-1.19)	1.13 (1.10-1.16)	1.11 (1.08-1.14)	1.12 (1.10-1.15)	1.09 (1.07-1.12)	1.08 (1.04-1.12)	1.10 (1.05-1.15)
**Gallbladder & biliary tract**	1.12 (1.05-1.18)	1.11 (1.05-1.18)	1.10 (1.05-1.16)	1.11 (1.06-1.16)	1.07 (1.02-1.12)	1.14 (1.04-1.23)	1.13 (1.01-1.25)
**Thyroid**	1.08 (1.04-1.13)	1.11 (1.06-1.17)	1.07 (1.02-1.13)	1.08 (1.04-1.12)	1.05 (1.02-1.09)	1.07 (0.99-1.16)	1.07 (0.97-1.18)
**Breast postmenopausal**	1.08 (1.06-1.09)	1.09 (1.08-1.11)	1.07 (1.05-1.09)	1.08 (1.06-1.09)	1.05 (1.04-1.07)	1.05 (1.03-1.08)	1.05 (1.02-1.08)
**Leukemia**	1.08 (1.05-1.11)	1.07 (1.03-1.11)	1.06 (1.04-1.09)	1.08 (1.05-1.10)	1.06 (1.04-1.09)	1.05 (1.01-1.09)	1.08 (1.01-1.14)
**Multiple myeloma**	1.07 (1.03-1.12)	1.07 (1.03-1.11)	1.08 (1.04-1.12)	1.08 (1.04-1.12)	1.06 (1.02-1.09)	1.07 (1.02-1.13)	1.09 (1.00-1.19)
**Testis**	1.05 (0.92-1.20)	1.07 (0.93-1.24)	1.04 (0.90-1.21)	1.04 (0.92-1.18)	1.03 (0.92-1.15)	1.06 (0.91-1.24)	1.09 (0.80-1.49)
**Brain and CNS**	1.05 (1.01-1.09)	1.08 (1.03-1.12)	1.05 (1.00-1.10)	1.06 (1.02-1.10)	1.04 (1.00-1.07)	1.06 (1.02-1.11)	1.03 (0.96-1.12)
**Colorectal**	1.04 (1.03-1.06)	1.06 (1.05-1.07)	1.05 (1.03-1.06)	1.05 (1.04-1.07)	1.03 (1.02-1.05)	1.05 (1.03-1.07)	1.05 (1.02-1.09)
**Hodgkin lymphoma**	1.04 (0.95-1.14)	1.03 (0.94-1.13)	1.08 (0.97-1.20)	1.07 (0.98-1.17)	1.05 (0.96-1.15)	1.04 (0.92-1.18)	1.12 (0.88-1.44)
**Liver**	1.03 (1.00-1.06)	1.04 (1.01-1.07)	1.05 (1.02-1.08)	1.06 (1.02-1.09)	1.05 (1.02-1.08)	1.05 (1.00-1.10)	1.10 (1.05-1.16)
**Ovary**	1.03 (0.98-1.08)	1.04 (0.99-1.10)	1.05 (1.00-1.09)	1.05 (1.01-1.09)	1.04 (1.01-1.08)	1.05 (0.99-1.12)	1.05 (0.94-1.17)
**Non-Hodgkin Lymph.**	1.02 (0.99-1.05)	1.05 (1.01-1.08)	1.04 (1.00-1.07)	1.04 (1.01-1.07)	1.02 (0.99-1.05)	1.04 (1.01-1.08)	1.04 (0.96-1.12)
**Malignant melanoma of skin**	1.01 (0.98-1.04)	1.03 (1.01-1.06)	1.01 (0.98-1.05)	1.02 (0.99-1.04)	1.00 (0.98-1.03)	1.01 (0.98-1.05)	0.98 (0.93-1.04)
**Cervix Uteri**	1.01 (0.95-1.07)	1.05 (0.96-1.15)	1.05 (0.98-1.12)	1.05 (0.98-1.13)	1.04 (0.99-1.09)	1.07 (0.99-1.16)	1.06 (0.97-1.16)

**Prostate**	1.01 (0.99-1.02)	1.03 (1.01-1.04)	1.00 (0.99-1.01)	1.01 (0.99-1.02)	0.99 (0.97-1.01)	0.99 (0.97-1.01)	0.97 (0.94-1.00)
**Bladder**	1.00 (0.98-1.02)	1.03 (1.01-1.04)	1.01 (0.99-1.04)	1.02 (1.00-1.03)	1.00 (0.98-1.02)	1.01 (0.99-1.04)	1.00 (0.95-1.05)
**Bone and articular cartilage**	0.99 (0.91-1.08)	1.04 (0.95-1.14)	1.04 (0.96-1.12)	1.03 (0.95-1.12)	1.01 (0.93-1.10)	1.02 (0.92-1.13)	1.03 (0.91-1.17)
**Pancreas**	0.98 (0.95-1.01)	1.00 (0.97-1.03)	1.01 (0.98-1.04)	1.01 (0.97-1.04)	1.00 (0.98-1.03)	1.03 (1.00-1.07)	1.07 (1.02-1.13)
**Breast premenopausal**	0.95 (0.91-0.99)	0.98 (0.94-1.03)	0.97 (0.92-1.02)	0.96 (0.91-1.00)	0.95 (0.90-1.00)	1.00 (0.93-1.08)	1.00 (0.91-1.09)
**Stomach**	0.91 (0.88-0.95)	0.94 (0.91-0.98)	0.99 (0.96-1.02)	0.97 (0.95-1.00)	0.99 (0.96-1.02)	1.02 (0.96-1.09)	1.07 (1.01-1.13)
**Head and Neck**	0.91 (0.88-0.94)	0.95 (0.92-0.98)	1.00 (0.97-1.03)	0.98 (0.95-1.01)	1.00 (0.97-1.03)	1.00 (0.96-1.03)	1.03 (0.95-1.11)
**Trachea, bronchus & Lung**	0.86 (0.85-0.88)	0.92 (0.90-0.94)	0.96 (0.94-0.97)	0.94 (0.92-0.96)	0.96 (0.93-0.98)	0.98 (0.96-1.00)	1.01 (0.98-1.04)
**Esophagus**	0.83 (0.78-0.88)	0.88 (0.82-0.93)	0.97 (0.90-1.05)	0.95 (0.89-1.02)	1.02 (0.96-1.08)	1.01 (0.94-1.08)	1.11 (0.95-1.31)
**Larynx**	0.82 (0.79-0.85)	0.89 (0.86-0.93)	0.95 (0.92-0.99)	0.92 (0.88-0.96)	0.95 (0.90-1.00)	0.95 (0.91-0.99)	1.02 (0.90-1.15)

Notes: Models are adjusted for geographic region of nationality, the MEDEA deprivation index, smoking status, and alcohol intake and stratified by age (5-year categories). Cumulative exposure is an exposure considering both degree and duration of overweight/obesity which is obtained by adding the difference between the BMI measurements that were ≥25 (≥30) kg/m^2 and 24.9 (29.9) kg/m^2 for every year lived with a BMI ≥25 and ≥30, respectively. Age of onset of a BMI ≥25 (and ≥30) kg/m^2 is only available for individuals who ever had a BMI ≥25 (≥30) kg/m^2 (N of cases are in Table S5) and the HRs of these exposures were inverted for visualization purposes (ie, an HR>1 means a greater risk at younger ages). Cancer types are ordered by descending ranking of the HRs for BMI at index date. The SD for each exposure were: 10 years for duration of BMI≥25 and 7 years of BMI≥30 kg/m^2, 69 cumulative overweight-years for cumulative exposure to a BMI≥25 and 36 cumulative obese-years to a BMI≥30kg/m^2, 7 years for age of onset of a BMI ≥25 and 8 years ≥30kg/m^2. Models for ovary, cervix, and corpus uteri cancers were only computed in females, for breast pre-menopausal only in pre-menopausal females, for breast post-menopausal only in post-menopausal females, and for prostate and testis only computed in males (their respective SDs can be consulted in Table S3). Brain and CNS includes pituitary gland and pineal gland tumors.

Abbreviations: BMI: Body Mass Index; CI: Confidence Interval; CNS: central nervous system; Cum: Cumulative; Lymph: lymphoma.

Table S3. Standard deviation of BMI at index date and other longitudinal BMI-derived exposures, by subgroups of participants

	Standard deviation for each subgroup of participants				
**Exposure**	**Overall population**	**Males**	**Females**	**Premenopausal females**	**Postmenopausal females**
**BMI at index**	4.89	4.48	5.23	5.33	5.11
**Duration of BMI ≥25 kg/m^2**	9.83	9.67	9.96	9.86	9.94
**Duration of BMI ≥25 kg/m^2**	6.98	6.42	7.42	6.79	7.63
**Cumulative exposure to BMI ≥25 kg/m^2**	68.83	61.03	74.86	69.57	76.58
**Cumulative exposure to BMI ≥30 kg/m^2**	35.50	28.38	40.6	36.80	41.97
**Age onset of BMI ≥25 kg/m^2**	6.91	6.91	6.90	7.19	6.78
**Age onset of BMI ≥30 kg/m^2**	7.57	7.38	7.64	7.65	7.63

Notes: These standard deviations were used to display the hazard ratios of Figure 1.
Abbreviations: BMI: Body mass index.

Figure S2. Hazard ratios of 26 cancer types related to BMI at index, with 95% CIs, allowing for non-linearity

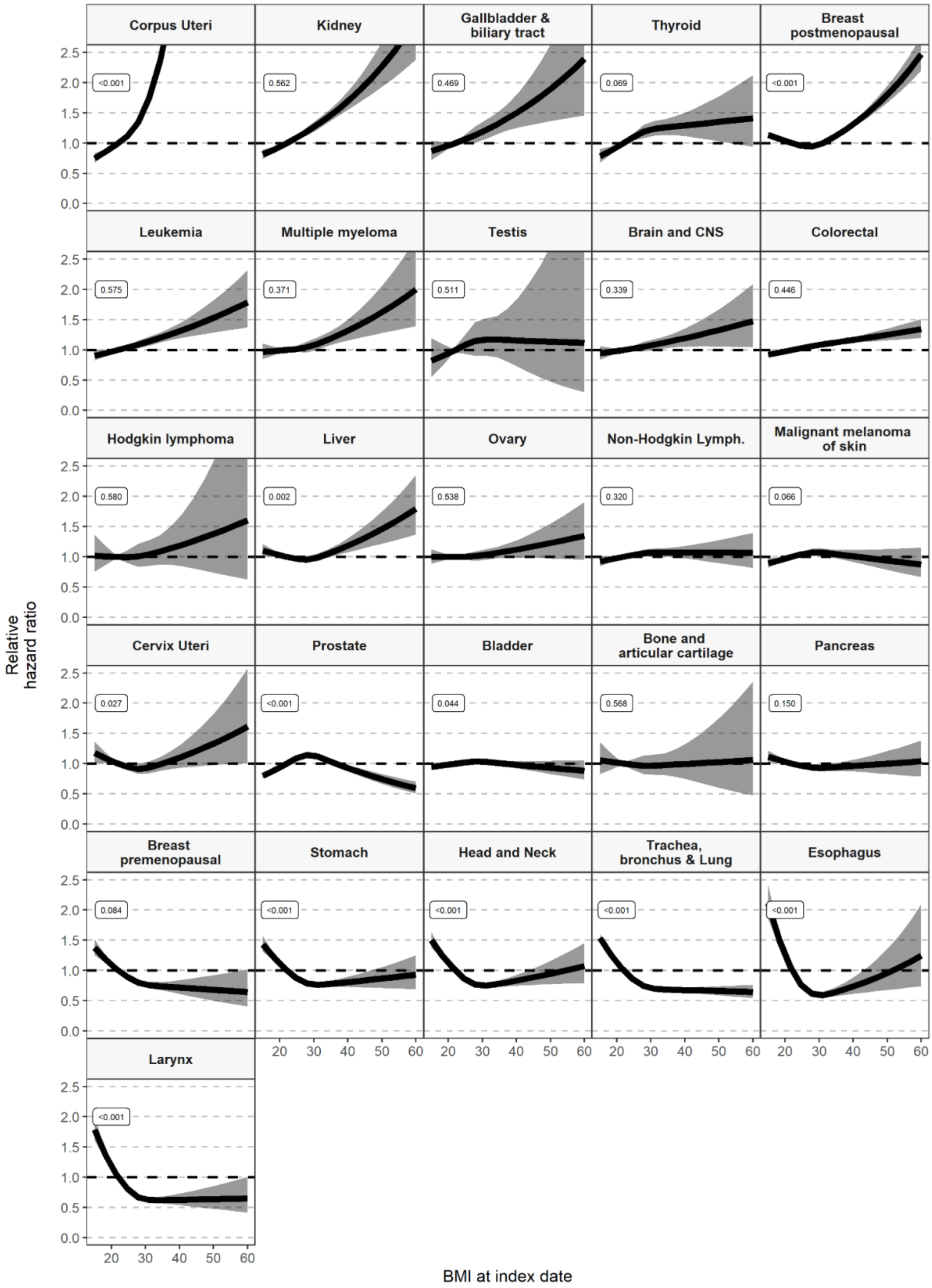

Notes: Data are presented as HRs with the respective 95% CIs. Source data are provided as a Source Data file. Models are adjusted for geographic region of nationality, the MEDEA deprivation index,

smoking status, and alcohol intake and stratified by age (5-year categories). These graphs were obtained using restricted cubic splines with 3 knots for BMI at index date with 22 kg/m^2 as the reference point. P-values for nonlinearity were obtained by comparing the model where BMI at index date was fitted with a nonlinear term against a linear model using a likelihood ratio test (two-sided without adjustment for multiple comparisons). Cancer types are ordered by descending ranking of the HRs for BMI at index date of Figure 1. Models for ovary, cervix, and corpus uteri cancers were only computed in females, for breast pre-menopausal only in pre-menopausal females, for breast post-menopausal only in post-menopausal females, and for prostate and testis only computed in males. Brain and CNS includes pituitary gland and pineal gland tumors.

Abbreviations: BMI: Body Mass Index; CI: Confidence Interval; CNS: central nervous system; Cum: Cumulative; Lymph: lymphoma.

Figure S3. Forest plot of hazard ratios of 26 cancer types related to one standard deviation increment of BMI at index date and other longitudinal BMI-derived exposures, minimally adjusted models, with 95% CIs

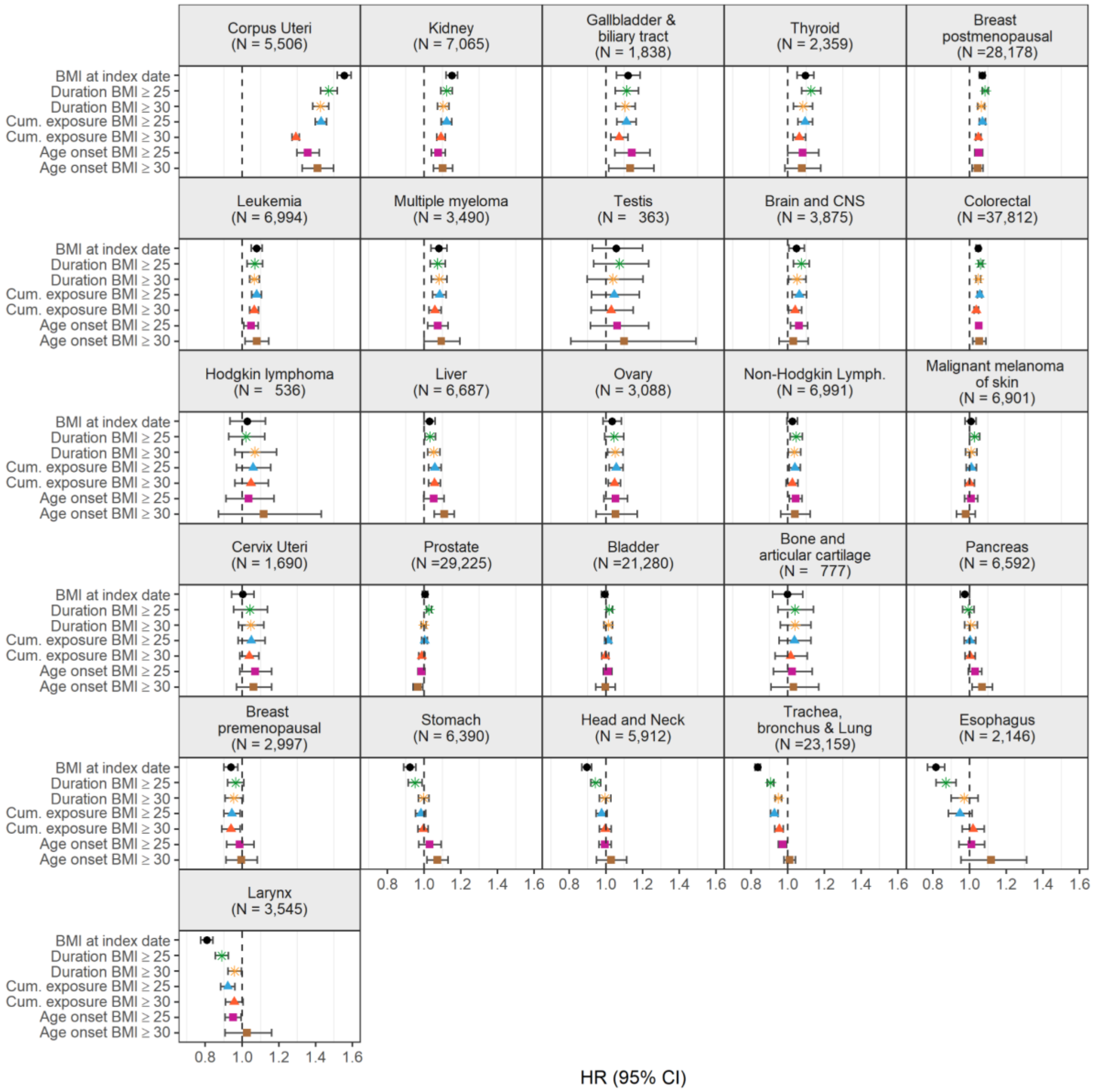

Notes: Data are presented as HRs (per one standard deviation increment) with the respective 95% CIs. Source data are provided as a Source Data file. The minimally-adjusted models included one exposure at a time and were adjusted by sex and stratified by age (5-year categories). Cumulative exposure is an exposure considering both degree and duration of overweight/obesity which is obtained by adding the difference between the BMI measurements that were ≥25 (≥30) kg/m^2 and 24.9 (29.9) kg/m^2 for every year lived with a BMI ≥25 and ≥30, respectively. Age of onset of a BMI ≥25 (and ≥30) kg/m^2 is only available for individuals who ever had a BMI ≥25 (≥30) kg/m^2 (N of cases are in Table S5) and the HRs of these exposures were inverted for visualization purposes (ie, an HR>1 means a greater risk at younger ages). Cancer types are ordered by descending ranking of the HRs for BMI at index date. The SD for each exposure were: 10 years for duration of BMI≥25 and 7 years of BMI≥30 kg/m^2, 69 cumulative overweight-years for cumulative exposure to a BMI≥25 and 36 cumulative obese-years to a BMI≥30kg/m^2, 7 years for age of onset of a BMI ≥25 and 8 years ≥30kg/m^2. Models for ovary, cervix, and corpus uteri cancers were only computed in females, for breast pre-menopausal only in pre-

menopausal females, for breast post-menopausal only in post-menopausal females, and for prostate and testis only computed in males (their respective SDs can be consulted in Table S3). Brain and CNS includes pituitary gland and pineal gland tumors.
Abbreviations: BMI: Body Mass Index; CI: Confidence Interval; CNS: central nervous system; Cum: Cumulative; HR: Hazard Ratio; Lymph: lymphoma.

Appendix 1. Description of the secondary and sensitivity analyses of this study

Associations observed in the main analyses were more pronounced among individuals aged <65 years for cancers of the *corpus uteri*, *kidney*, *thyroid*, *leukemia*, *liver*, and *head and neck* cancers, compared to those ≥65 years; and among males for *gallbladder and biliary tract, stomach*, and *respiratory tract* cancers, compared to females (Figure S4). When we mutually adjusted the models for age of onset of a BMI ≥25 (≥30) and duration of BMI ≥25 (≥30) most associations became null; however, these results are not straightforward to interpret due to the strong collinearity between these exposures (eg, correlation between duration and age of onset of BMI≥25: 0.94) that may lead to instability of the coefficients (Figures S5, S6). The inverse associations (for *stomach* and respiratory tract cancers) became null when we restricted the analyses to never smokers. Moreover, BMI at index date, duration of, and cumulative exposure to a BMI ≥25 (≥30) became positively and more pronouncedly, respectively, associated with *head and neck* and *bladder* cancers (Figure S7). Overall, the changes in risk discrimination after adding longitudinal exposures to the fully-adjusted models with BMI at index date as the main exposure were modest (all differences in Harrell's C-index between -0.03 to 0.03) (Figure S8). The associations with the recalculated exposures (restricted to BMIs ≥25 and <30 kg/m^2 to investigate the independent effect of overweight from obesity in relation to cancer risk) were more attenuated than those of the main analyses (where we analyzed any BMIs ≥25) (Figure S9). Also, several statistically-significant associations in the main analysis became null in this secondary one (8 out of 14 for duration of, 5 out of 13 for cumulative exposure to, and 3 out of 13 for age of onset of a BMI ≥25 and <30 kg/m^2). However, the point estimates of the associations that became null were all ≥1 (eg, HR of *gallbladder and biliary tract* for duration of BMI ≥25 in the main analysis: 1.11 [1.05-1.18] vs for duration of BMI ≥25 and <30 in the secondary analysis 1.03 [0.98-1.09]) (Figure S9).

Overall, our results were similar to those from five sensitivity analyses (Figure S10 and Figure S11). However, there were some differences in the analyses restricted to individuals with at least one BMI assessment in their health records, for whom the inverse associations or trends observed in the main analysis for *breast premenopausal* or respiratory tract cancers, respectively, became stronger (eg, HR of *trachea, bronchus, and lung* for BMI at baseline in the main analysis: 0.86 [0.85-0.88] vs in the sensitivity analysis 0.81 [0.79-0.82]) (Figure S10). Further, when we applied the Bonferroni correction to counteract the fact that we were testing multiple comparisons, some of the positive associations of the main analysis became null (Figure S11). Longer duration of a BMI≥30 kg/m^2 was positively associated with the risk of 9 cancers in the sensitivity analysis instead of 12 in the main analysis (affecting *non-Hodgkin lymphoma* and cancers of the *brain and the CNS* and *liver*). Higher cumulative exposure to a BMI≥25 kg/m^2 with 12 instead of 13 cancers (no longer with *bladder* cancer). Higher cumulative exposure to a BMI≥30 kg/m^2 with 10 instead of 11 cancers (no longer with *brain and the CNS* cancer). Age of onset of a BMI≥25 kg/m^2 with 8 instead of 11 cancers (no longer with *leukemia*, and cancers of the *liver* and *pancreas*). Age of onset of a BMI≥30 kg/m^2 with 6 instead of 10 cancers (no longer with *leukemia*, *multiple myeloma*, and cancers of the *stomach* and *gallbladder and biliary tract*). Finally, BMI at index date with 8 instead of 10 cancers (no longer with cancers of the *brain and the CNS* and *liver*) (Figure 1, Figure S11).

Figure S4. Hazard ratios of 26 cancer types related to one standard deviation increment of BMI at index date and other longitudinal BMI-derived exposures stratified by age and sex, with 95% CIs

A. Age

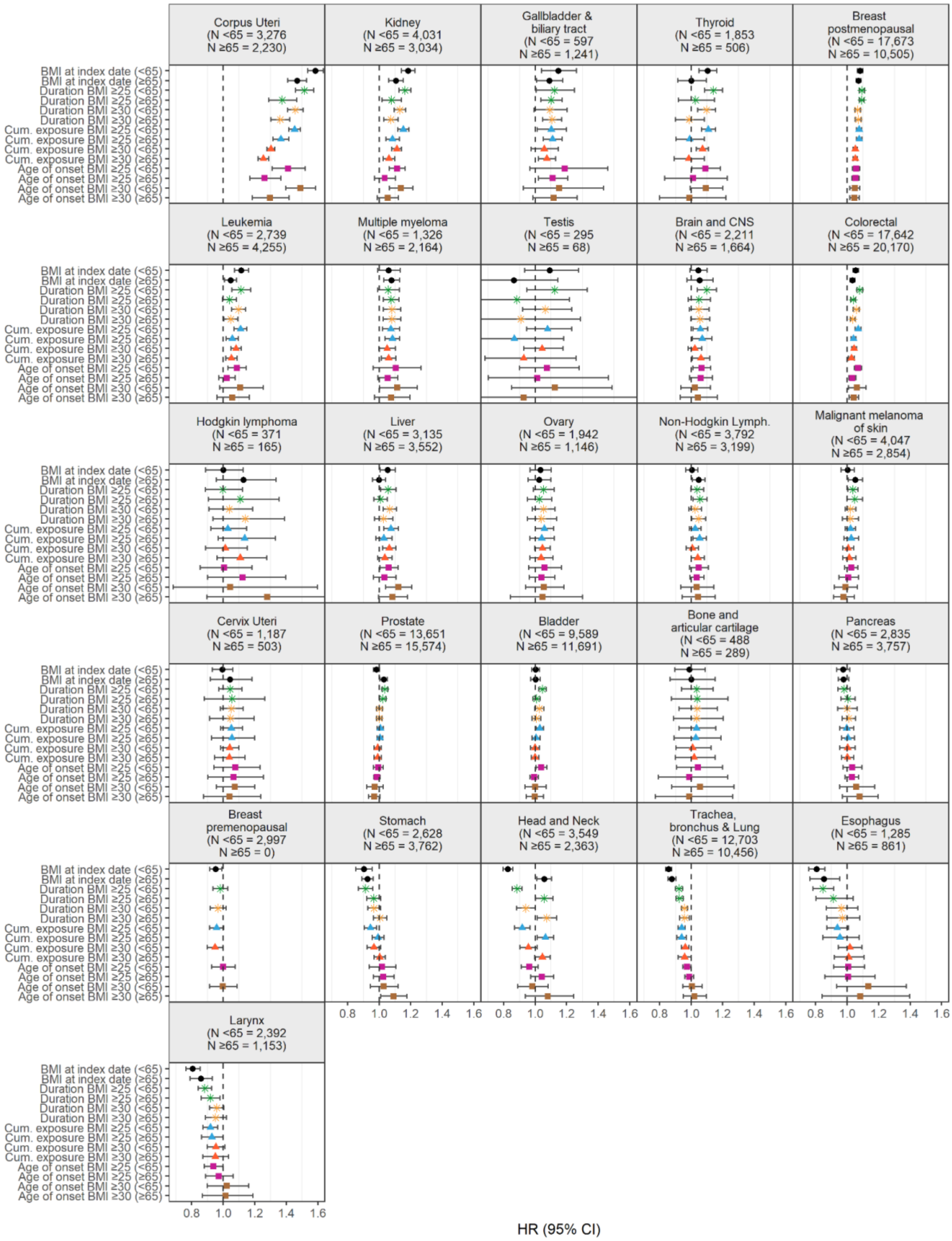

B. Sex

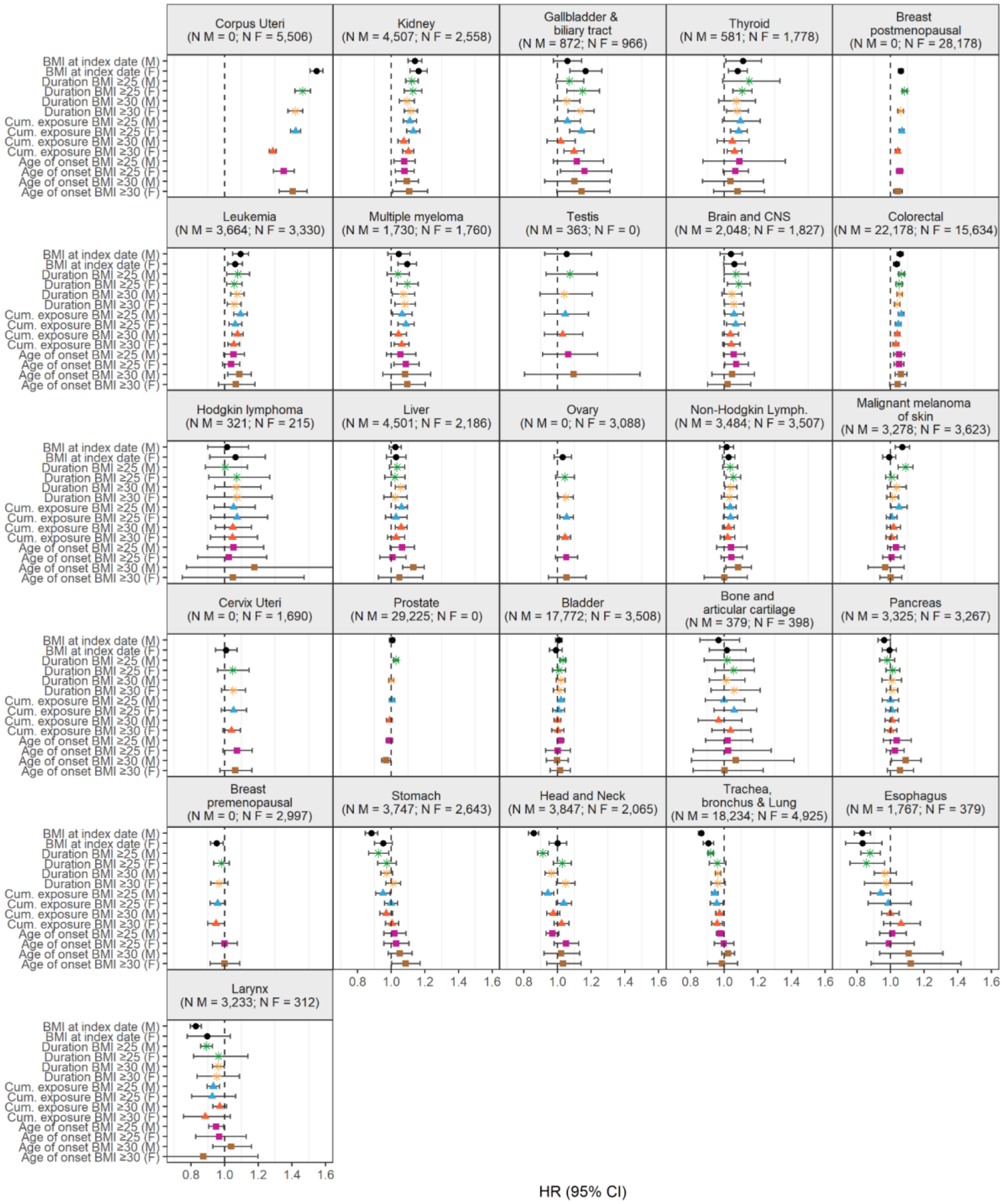

Notes: Data are presented as HRs (per one standard deviation increment) with the respective 95% CIs. Source data are provided as a Source Data file. Models are adjusted for geographic region of nationality, the MEDEA deprivation index, smoking status, and alcohol intake and stratified by age (5-year categories). Cumulative exposure is an exposure considering both degree and duration of overweight/obesity which is obtained by adding the difference between the BMI measurements that were ≥25 (≥30) kg/m^2 and 24.9 (29.9) kg/m^2 for every year lived with a BMI ≥25 and ≥30, respectively. Age of onset of a BMI ≥25 (and ≥30) kg/m^2 is only available for individuals who ever had a BMI ≥25 (≥30) kg/m^2 (N of cases are in Table S5) and the HRs of these exposures were inverted for visualization purposes (ie, an HR>1 means a greater risk at younger ages). Cancer types are ordered by descending

ranking of the HRs for BMI at index date. Models for ovary, cervix, and corpus uteri cancers were only computed in females, for breast pre-menopausal only in pre-menopausal females, for breast post-menopausal only in post-menopausal females, and for prostate and testis only computed in males. Brain and CNS includes pituitary gland and pineal gland tumors.

Abbreviations: BMI: Body Mass Index; CI: Confidence Interval; CNS: central nervous system; Cum: Cumulative; F: Females; HR: Hazard Ratio; Lymph: lymphoma; M: Males.

Figure S5. Hazard ratios of 26 cancer types related to one standard deviation increment of BMI at index date and other longitudinal BMI-derived exposures with mutual adjustment of duration of BMI ≥25 (≥30) kg/m^2 and age of onset of BMI ≥25 (≥30) kg/m^2, respectively, with 95% CIs

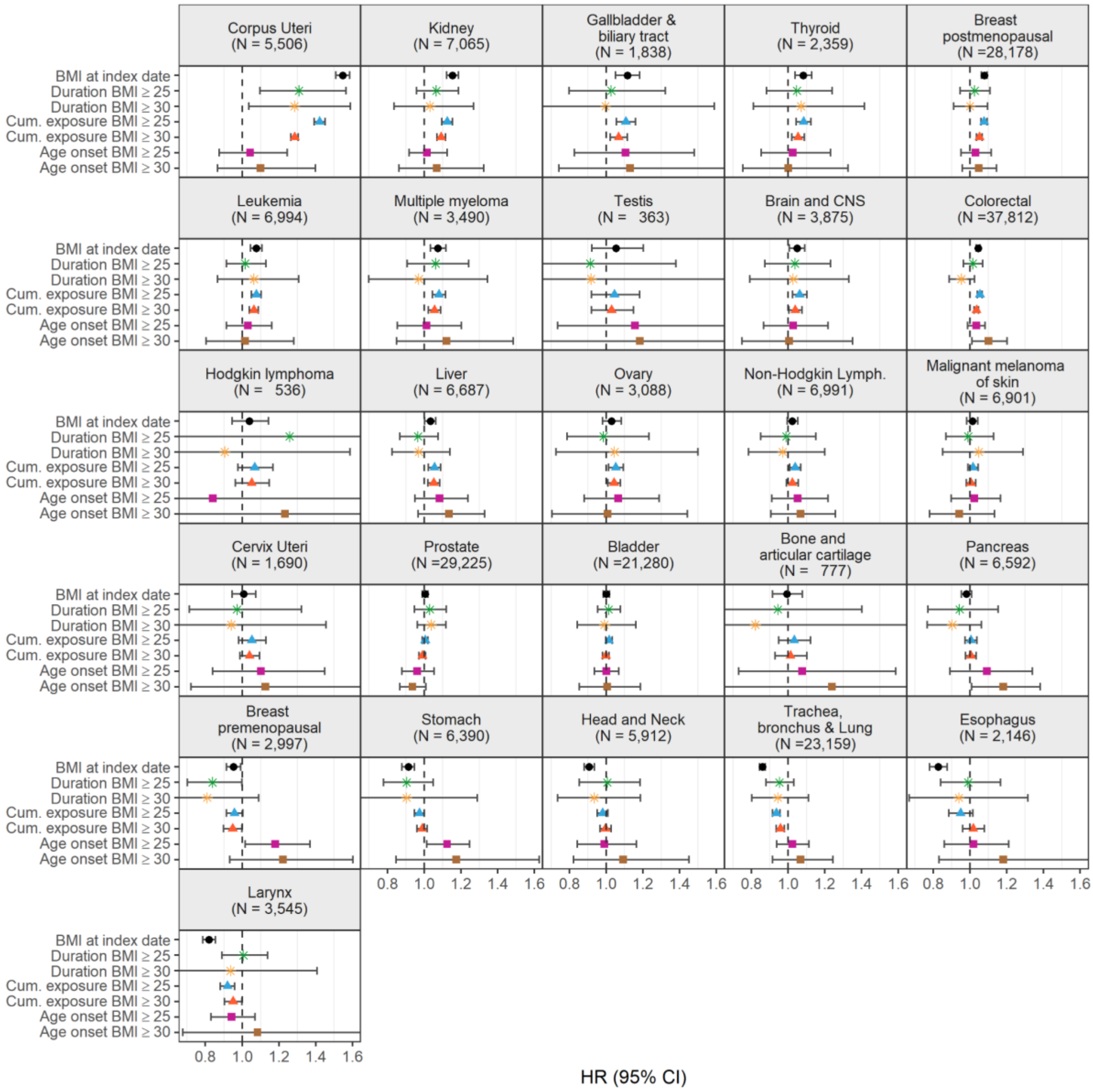

Notes: Data are presented as HRs (per one standard deviation increment) with the respective 95% CIs. Source data are provided as a Source Data file. All models are adjusted for geographic region of nationality, the MEDEA deprivation index, smoking status, and alcohol intake and stratified by age (5-year categories). Moreover, we mutually-adjusted the models including age of onset of a BMI ≥25 (and ≥30) kg/m^2 and duration of BMI ≥25 (and ≥30) kg/m^2 to distinguish the effect of age of onset from that of the duration of years exposed to overweight/obesity. Thus the number of individuals included in the models of duration of BMI ≥25 and age of onset of a BMI ≥25 corresponds to those who ever had a BMI ≥25; and that of duration of BMI ≥30 and age of onset of a BMI ≥30 corresponds to those who ever had a BMI ≥30 (N of cases are in Table S5). The HRs of age of onset were inverted for visualization purposes (ie, an HR>1 means a greater risk at younger ages). Cancer types are ordered by descending ranking of the HRs for BMI at index date. Models for ovary, cervix, and corpus uteri cancers were only computed in females, for breast pre-menopausal only in pre-menopausal females, for breast post-menopausal only in post-menopausal females, and for prostate and testis only computed in males. Brain and CNS includes pituitary gland and pineal gland tumors.

Abbreviations: BMI: Body Mass Index; CI: Confidence Interval; CNS: central nervous system; Cum: Cumulative; HR: Hazard Ratio; Lymph: lymphoma.

Figure S6. Pearson's correlation matrix between exposures

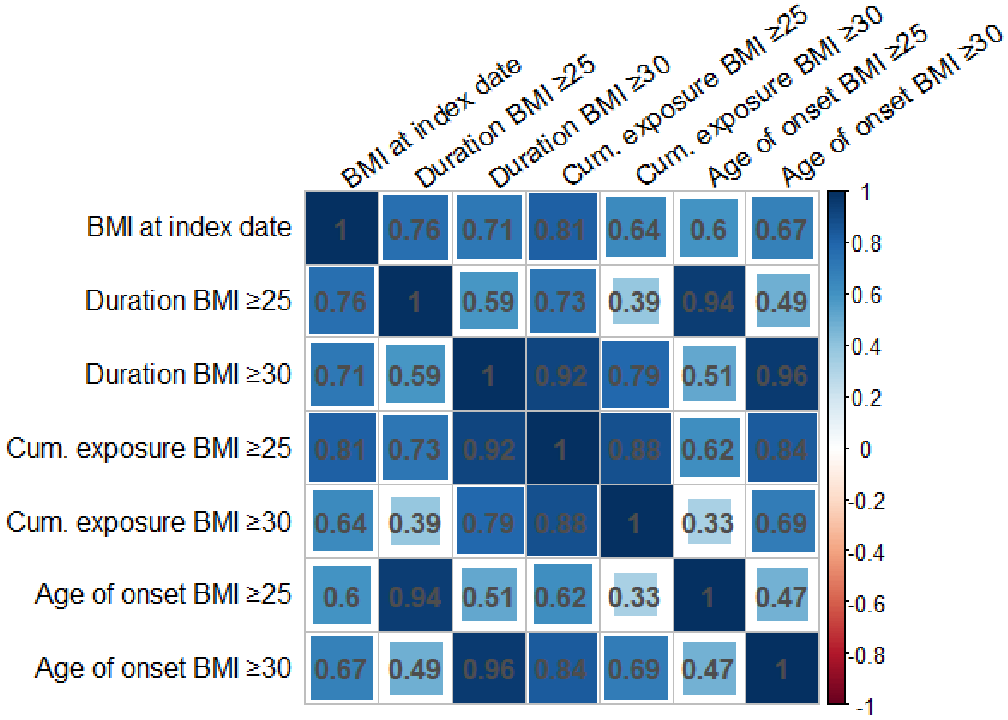

Abbreviations: BMI: body mass index; Cum: cumulative.

Figure S7. Hazard ratios of 26 cancer types related to one standard deviation increment of BMI at index date and other longitudinal BMI-derived exposures restricted to never smokers, with 95% CIs

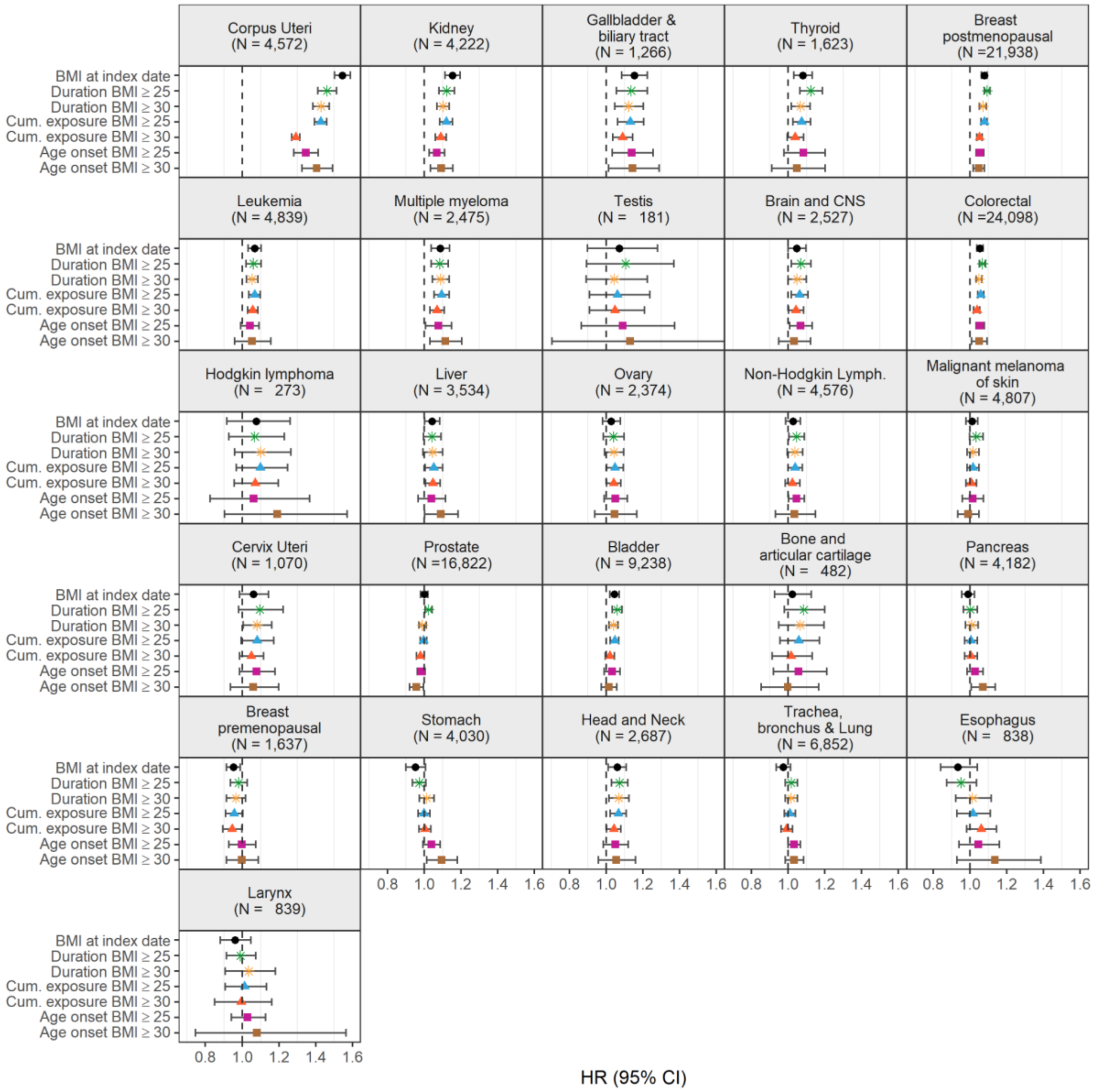

Notes: Data are presented as HRs (per one standard deviation increment) with the respective 95% CIs. Source data are provided as a Source Data file. Models are adjusted for geographic region of nationality, the MEDEA deprivation index, smoking status, and alcohol intake and stratified by age (5-year categories). Cumulative exposure is an exposure considering both degree and duration of overweight/obesity which is obtained by adding the difference between the BMI measurements that were ≥25 (≥30) kg/m^2 and 24.9 (29.9) kg/m^2 for every year lived with a BMI ≥25 and ≥30, respectively. Age of onset of a BMI ≥25 (and ≥30) kg/m^2 is only available for individuals who ever had a BMI ≥25 (≥30) kg/m^2 (N of cases are in Table S5) and the HRs of these exposures were inverted for visualization purposes (ie, an HR>1 means a greater risk at younger ages). Cancer types are ordered by descending ranking of the HRs for BMI at index date. Models for ovary, cervix, and corpus uteri cancers were only computed in females, for breast pre-menopausal only in pre-menopausal females, for breast post-menopausal only in post-menopausal females, and for prostate and testis only computed in males. Brain and CNS includes pituitary gland and pineal gland tumors.

Abbreviations: BMI: Body Mass Index; CI: Confidence Interval; CNS: central nervous system; Cum: Cumulative; HR: Hazard Ratio; Lymph: lymphoma.

Figure S8. Changes in risk discrimination for the risk of 26 cancer types after addition of longitudinal BMI-derived exposures to the fully-adjusted model including BMI at index date as the main exposure

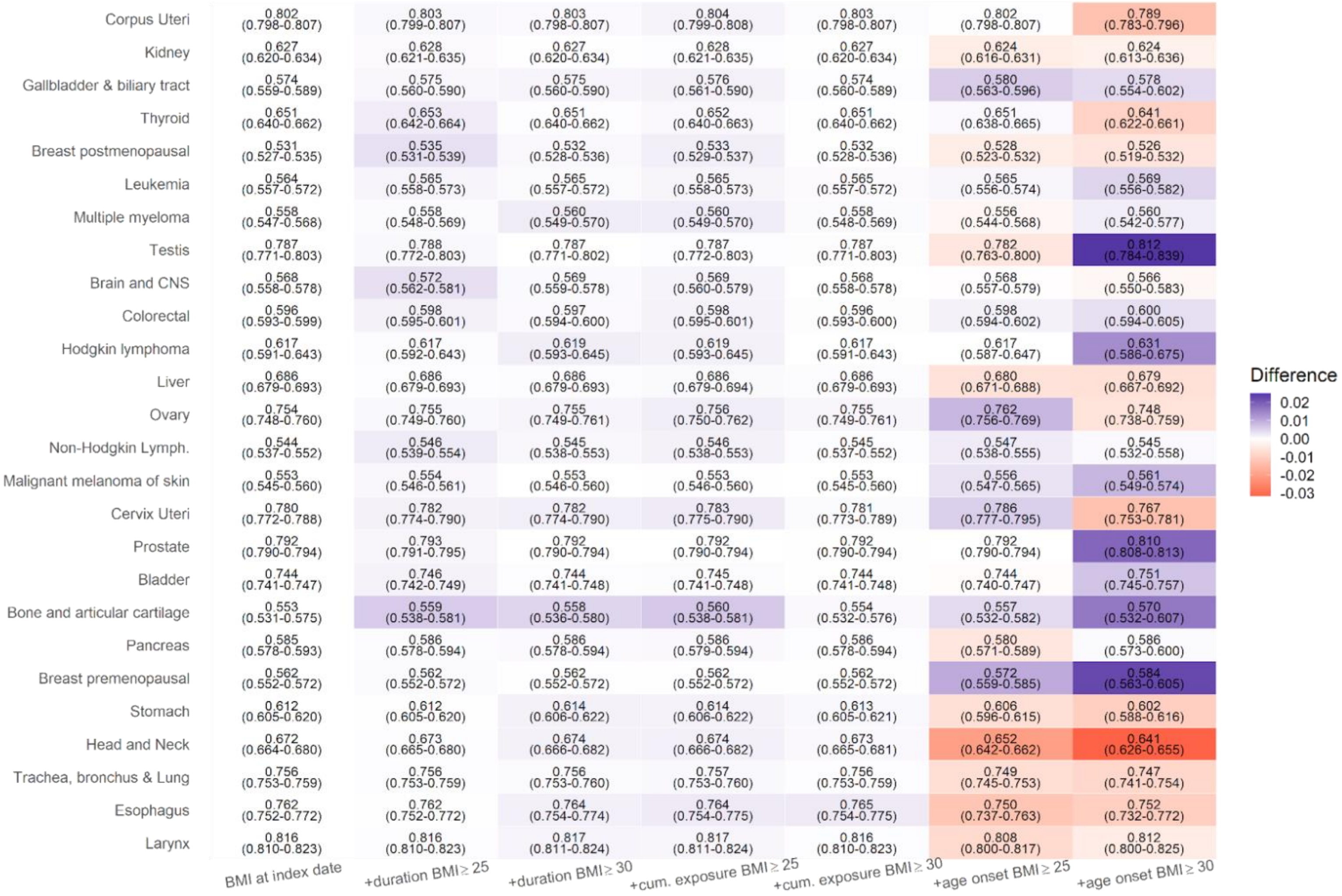

Notes: Difference in Harrell's C-index scores between the main models (adjusted by sex, geographic region of nationality, the MEDEA deprivation index, smoking status, and alcohol intake and stratification by age in 5-year categories) including BMI at index as the main exposure (first column) and other models that further included each of the longitudinal exposures separately (one type of model per column). Negative differences (red cells) represent a worsen discrimination with respect to the model with BMI at baseline, positive differences (green cells) represent an improved discrimination. Cancer types are ordered by descending ranking of the HRs for BMI at index date. Models for ovary, cervix, and corpus uteri cancers were only computed in females, for breast pre-menopausal only in pre-menopausal females, for breast post-menopausal only in post-menopausal females, and for prostate and testis only computed in males. Brain and CNS include pituitary gland and pineal gland tumors.
Abbreviations: BMI: Body Mass Index; CNS: Central nervous system; CI:confidence interval; cum: cumulative; lymph: lymphoma.

Figure S9. Hazard ratios of 26 cancer types related to one standard deviation increment of BMI at index date and other longitudinal BMI-derived exposures restricted to BMIs ≥25 and <30 kg/m², with 95% CIs

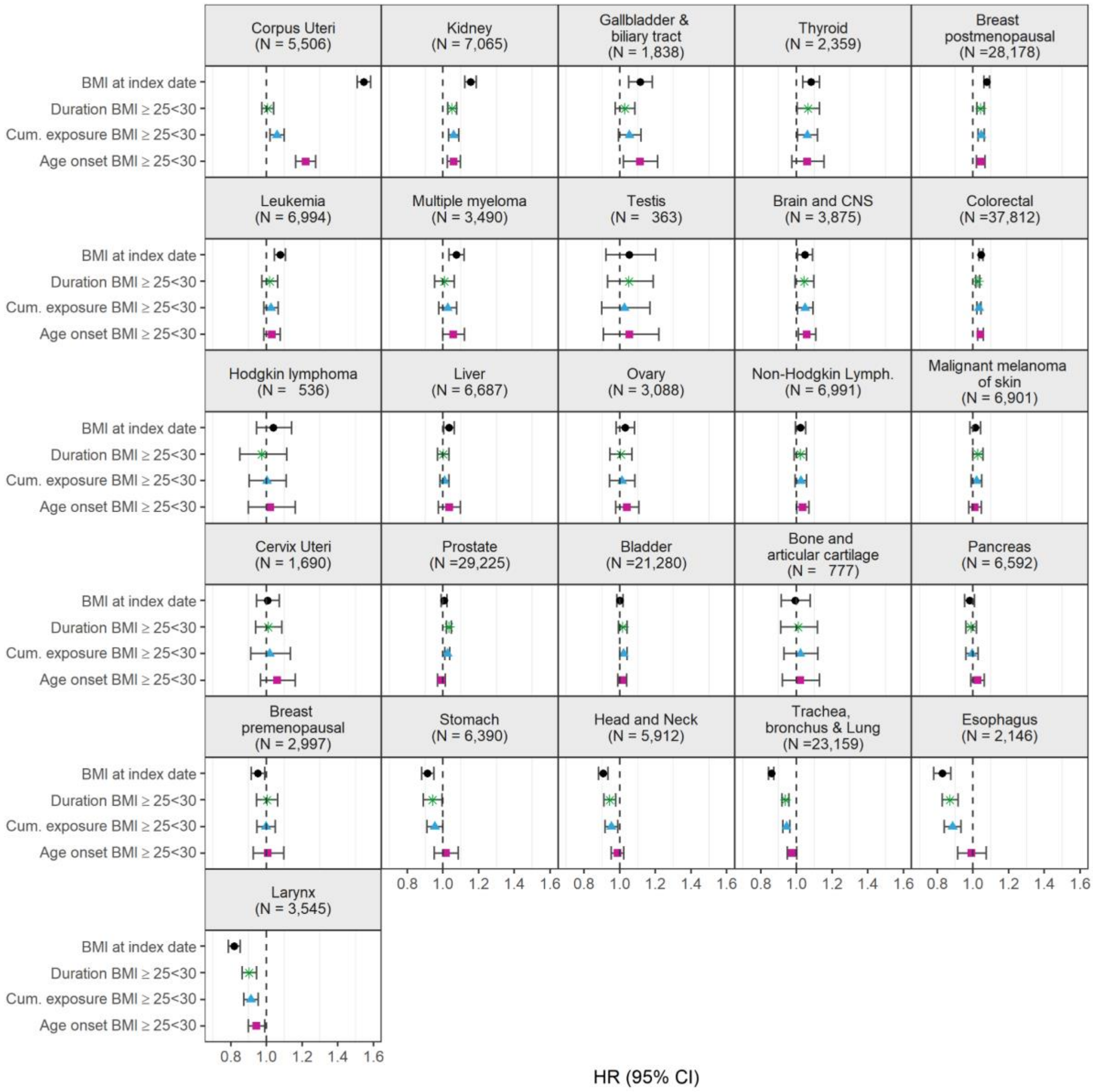

Notes: Data are presented as HRs (per one standard deviation increment) with the respective 95% CIs. Source data are provided as a Source Data file. Models are adjusted for geographic region of nationality, the MEDEA deprivation index, smoking status, and alcohol intake and stratified by age (5-year categories). Cumulative exposure is an exposure considering both degree and duration of overweight/obesity which is obtained by adding the difference between the BMI measurements that were ≥25 and <30 kg/m² and 24.9 and <30 kg/m² for every year lived with a BMI ≥25 and <30, respectively. Age of onset of a BMI ≥25 <30 kg/m² is only available for individuals who ever had a BMI ≥25 and <30 kg/m² (N of cases are in Table S5) and the HRs of this exposure were inverted for visualization purposes (ie, an HR>1 means a greater risk at younger ages). Cancer types are ordered by descending ranking of the HRs for BMI at index date. Models for ovary, cervix, and corpus uteri cancers were only computed in females, for breast pre-menopausal only in pre-menopausal females, for breast post-menopausal only in post-menopausal females, and for prostate and testis only computed in males. Brain and CNS includes pituitary gland and pineal gland tumors.

Abbreviations: BMI: Body Mass Index; CI: Confidence Interval; CNS: central nervous system; Cum: Cumulative; HR: Hazard Ratio; Lymph: lymphoma.

Figure S10. Hazard ratios of 26 cancer types related to one standard deviation increment of BMI at index date and other longitudinal BMI-derived exposures in sensitivity analyses, with 95% CIs

A. Adjustment by difference in BMI at index and end of the longitudinal-exposure window

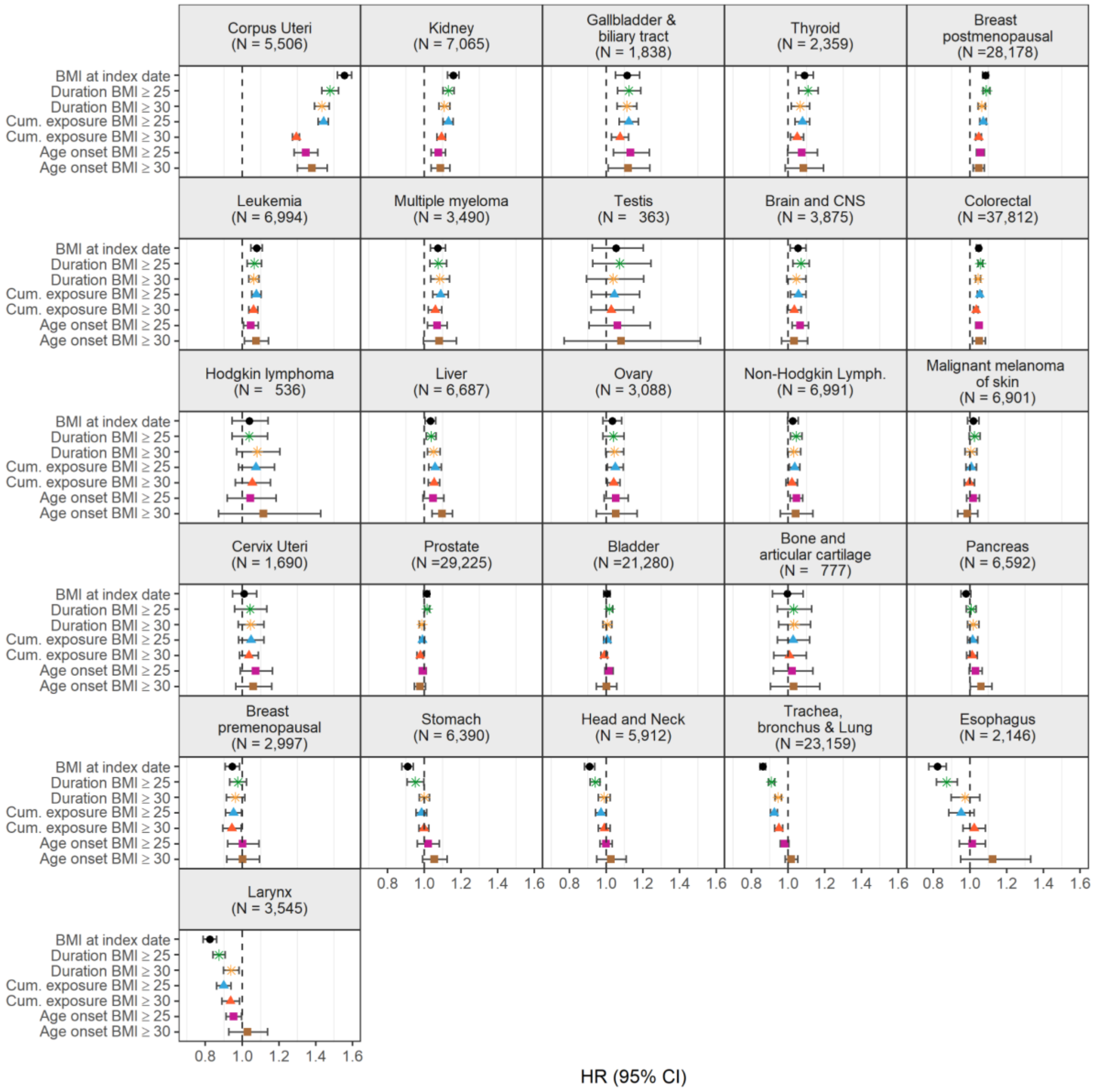

B. Restricted to individuals with at least 1 BMI assessment in their EHRs

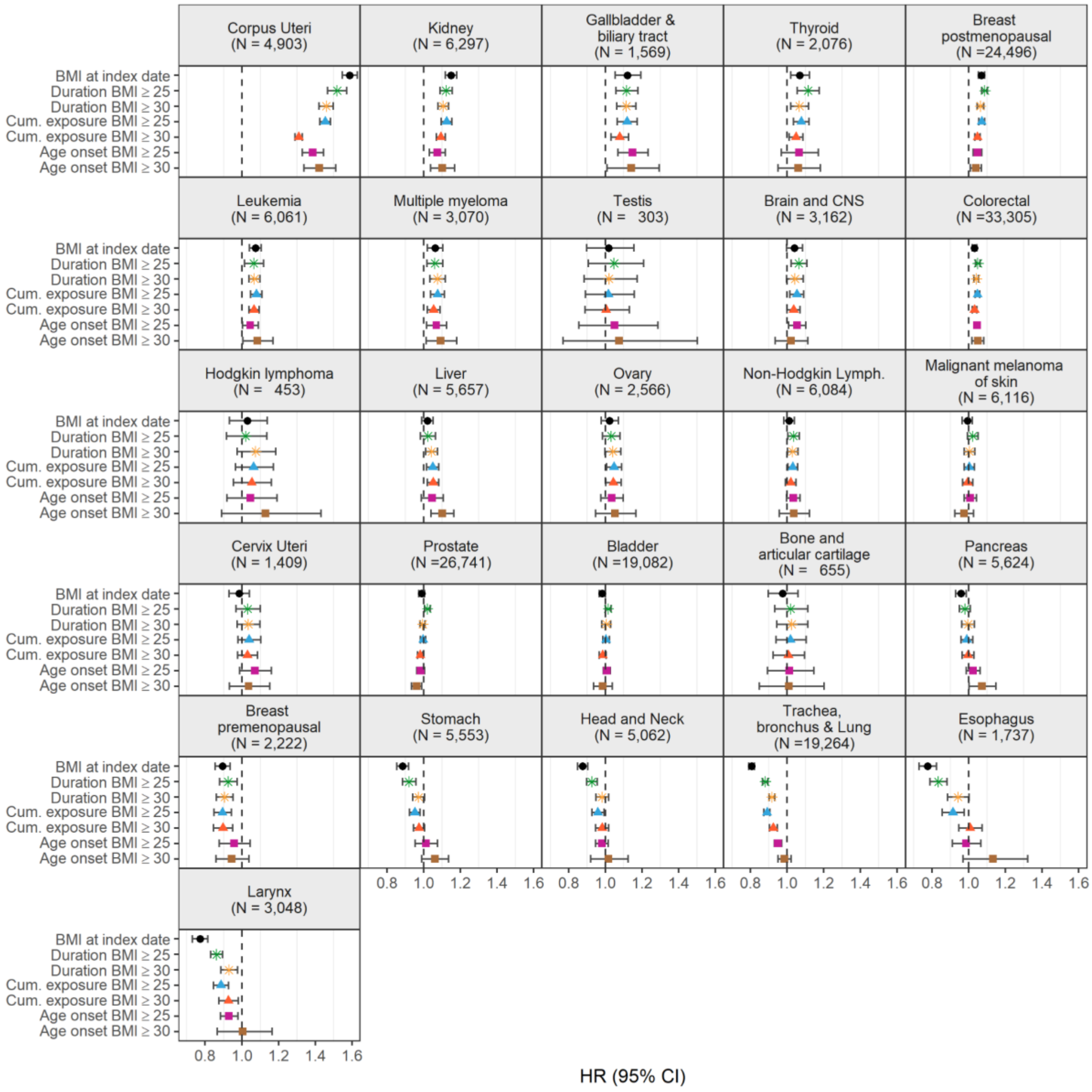

C. Start of follow up 3 years after index date (instead of 1 year)

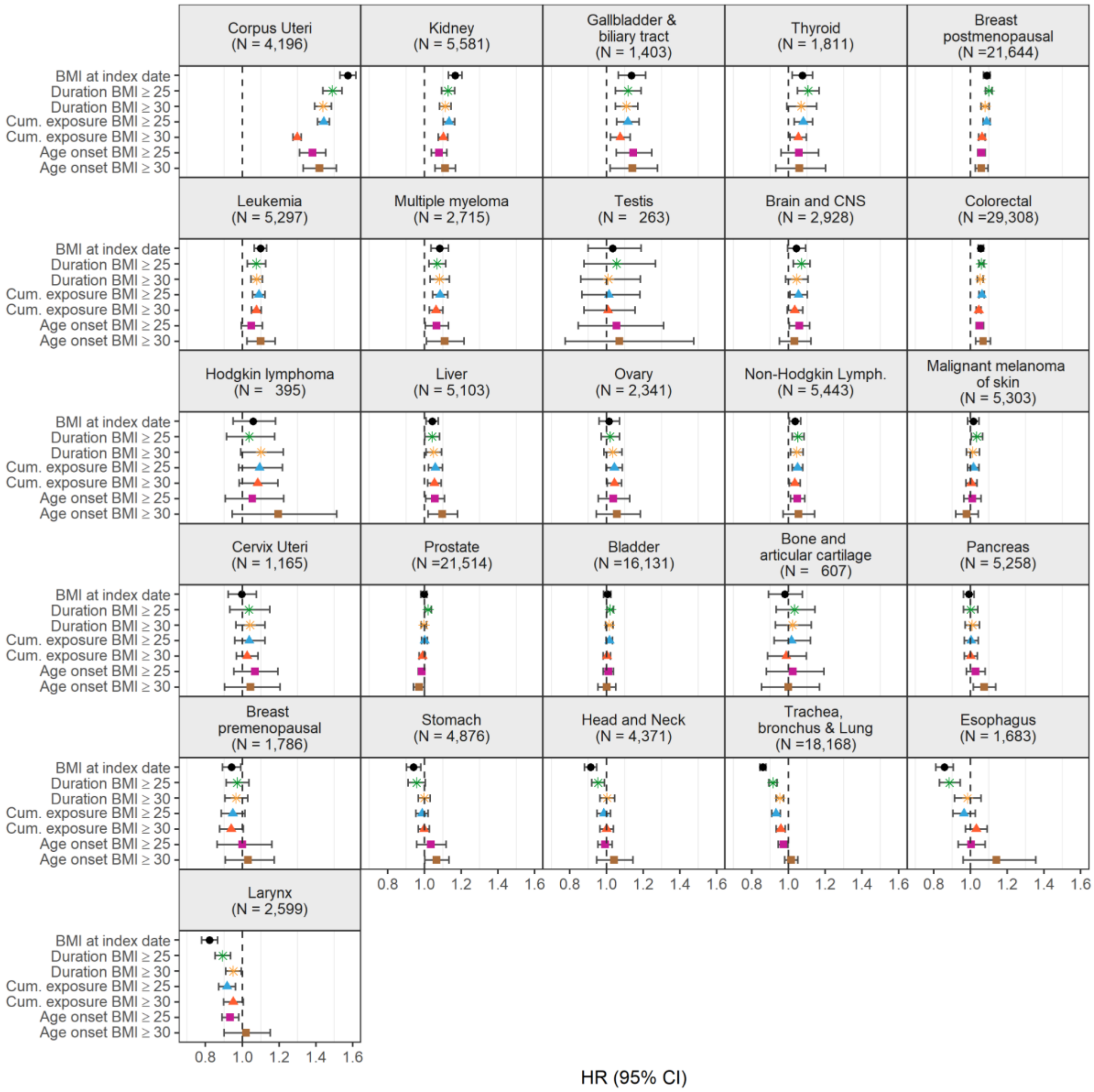

Notes: Data are presented as HRs (per one standard deviation increment) with the respective 95% CIs. Source data are provided as a Source Data file. Models are adjusted for geographic region of nationality, the MEDEA deprivation index, smoking status, and alcohol intake and stratified by age (5-year categories). Cumulative exposure is an exposure considering both degree and duration of overweight/obesity which is obtained by adding the difference between the BMI measurements that were ≥25 (≥30) kg/m^2 and 24.9 (29.9) kg/m^2 for every year lived with a BMI ≥25 and ≥30, respectively. Age of onset of a BMI ≥25 (and ≥30) kg/m^2 is only available for individuals who ever had a BMI ≥25 (≥30) kg/m^2 (N of cases are in Table S5) and the HRs of these exposures were inverted for visualization purposes (ie, an HR>1 means a greater risk at younger ages). Cancer types are ordered by descending ranking of the HRs for BMI at index date. Models for ovary, cervix, and corpus uteri cancers were only computed in females, for breast pre-menopausal only in pre-menopausal females, for breast post-menopausal only in post-menopausal females, and for prostate and testis only computed in males. Brain and CNS includes pituitary gland and pineal gland tumors.

Abbreviations: BMI: Body Mass Index; CI: Confidence Interval; CNS: central nervous system; Cum: Cumulative; HR: Hazard Ratio; Lymph: lymphoma.

Figure S11. Hazard ratios of 26 cancer types related to one standard deviation increment of BMI at index date and other longitudinal BMI-derived exposures in sensitivity analyses, with Bonferroni correction

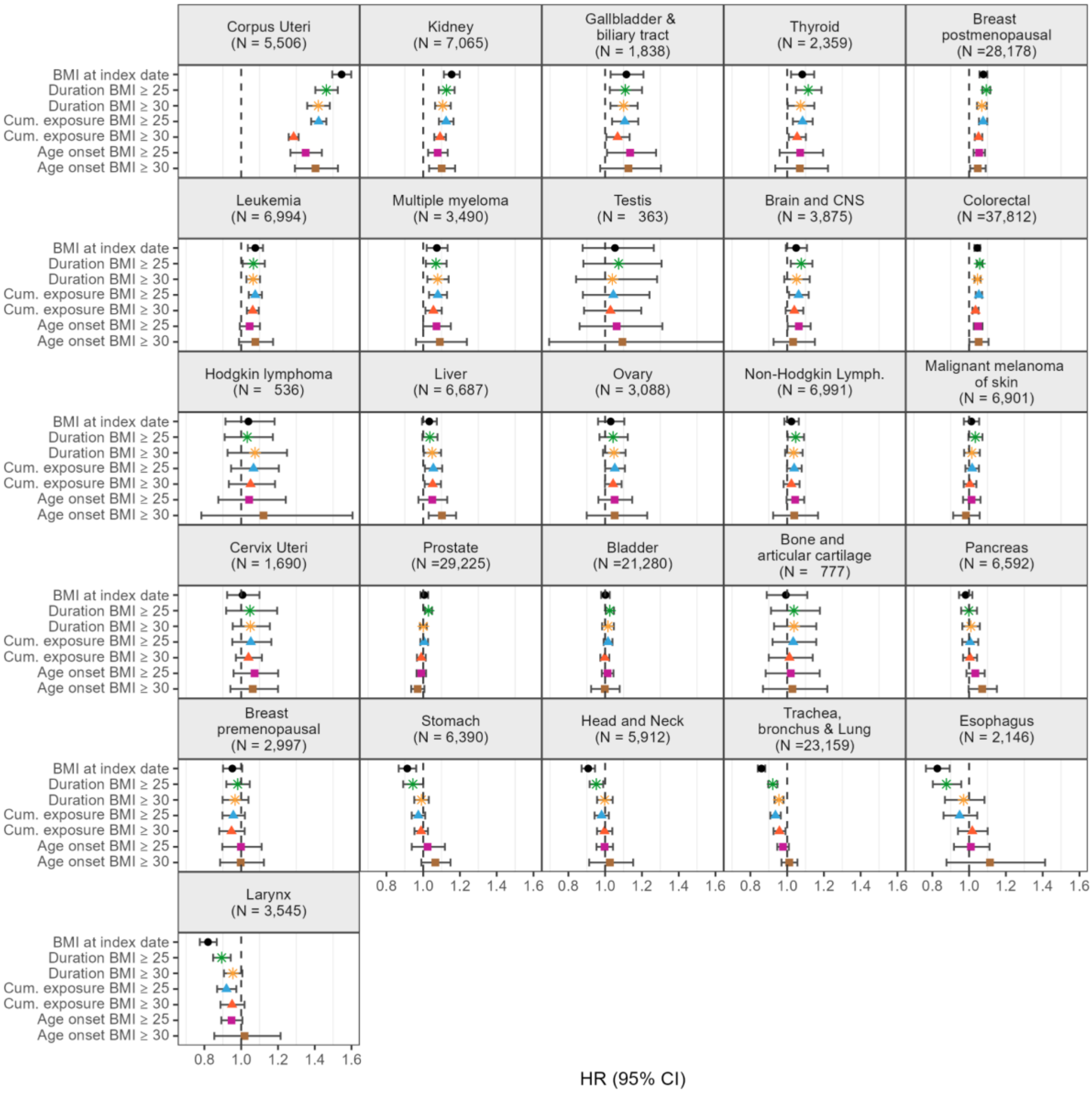

Notes: Data are presented as HRs (per one standard deviation increment) with the respective 95% CIs. Source data are provided as a Source Data file. Models are adjusted for geographic region of nationality, the MEDEA deprivation index, smoking status, and alcohol intake and stratified by age (5-year categories). Cumulative exposure is an exposure considering both degree and duration of overweight/obesity which is obtained by adding the difference between the BMI measurements that were ≥25 (≥30) kg/m^2 and 24.9 (29.9) kg/m^2 for every year lived with a BMI ≥25 and ≥30, respectively. Age of onset of a BMI ≥25 (and ≥30) kg/m^2 is only available for individuals who ever had a BMI ≥25 (≥30) kg/m^2 (N of cases are in Table S5) and the HRs of these exposures were inverted for visualization purposes (ie, an HR>1 means a greater risk at younger ages). Cancer types are ordered by descending ranking of the HRs for BMI at index date. The SD for each exposure were: 10 years for duration of BMI≥25 and 7 years of BMI≥30 kg/m^2, 69 cumulative overweight-years for cumulative exposure to a BMI≥25 and 36 cumulative obese-years to a BMI≥30kg/m^2, 7 years for age of onset of a BMI ≥25 and 8 years ≥30kg/m^2. Models for ovary, cervix, and corpus uteri cancers were only computed in females, for breast pre-menopausal only in pre-menopausal females, for breast post-menopausal only in post-menopausal females, and for prostate and testis only computed in males (their respective SDs can be consulted in Table S3). Brain and CNS includes pituitary gland and pineal gland tumors.

Abbreviations: BMI: Body Mass Index; CI: Confidence Interval; CNS: central nervous system; Cum: Cumulative; HR: Hazard Ratio; Lymph: lymphoma.

Figure S12. Summary of the findings of this study and the Viewpoint of the IARC Working Group on Body Fatness and Cancer.

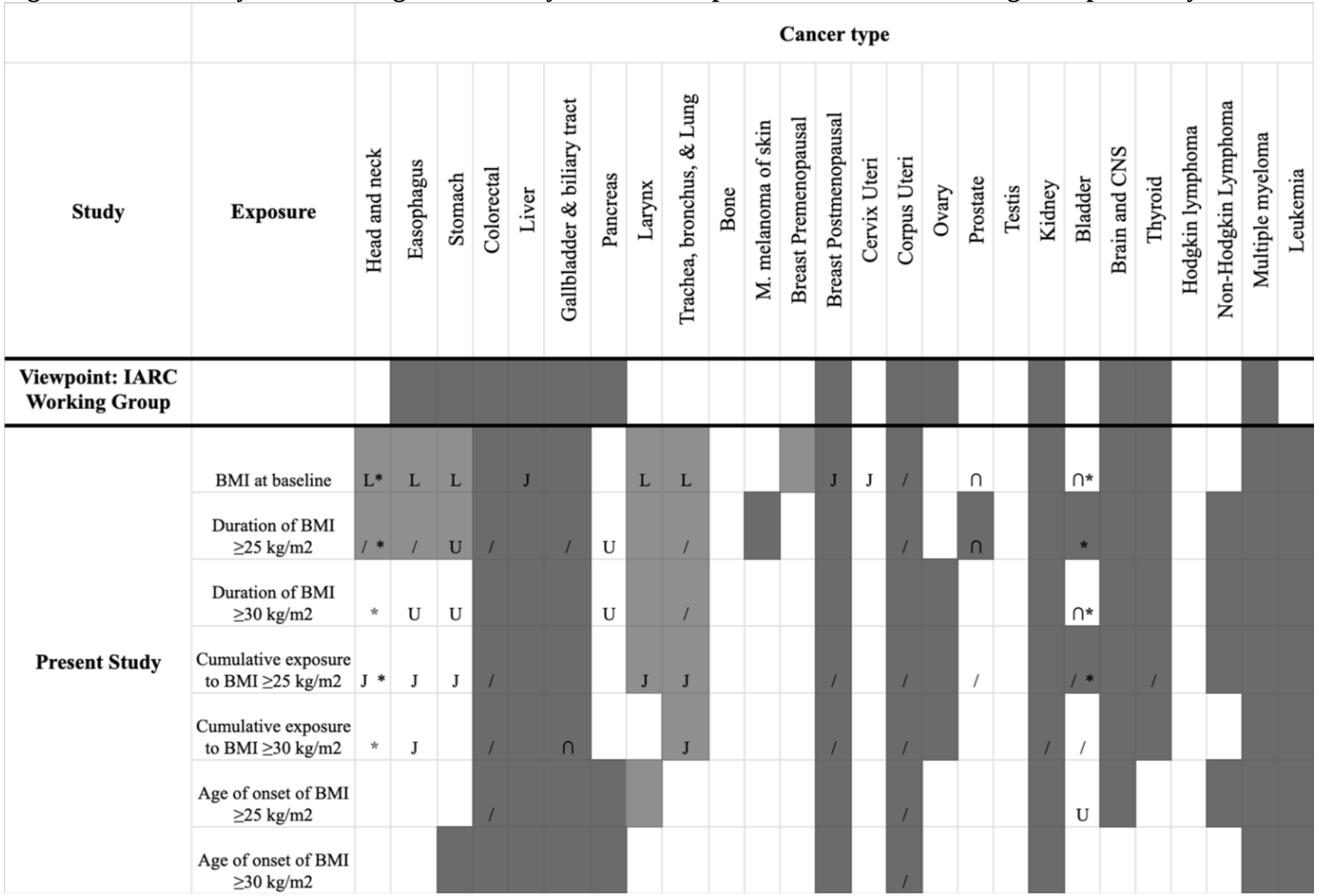

Notes: Own elaboration with data from the present study and Lauby-Secretan B, Scoccianti C, Loomis D, Grosse Y, Bianchini F, Straif K. Body Fatness and Cancer — Viewpoint of the IARC Working Group. New England Journal of Medicine. 2016;375(8):794-798. doi:10.1056/NEJMsr1606602. Cells filled in dark grey denote positive linear associations and lighter grey denote negative linear associations. Letters in the intersection between exposures and cancer types represent the shape of observed non-linear associations. Cells marked with "L" represent L-shaped associations between the exposures and specific cancer types. Cells marked with "J" represent J-shaped associations between the exposures and specific cancer types. Cells marked with "∩" represent inverted-U-shaped associations between the exposures and specific cancer types. Cells marked with "U" represent U-shaped associations between the exposures and specific cancer

types. Cells marked with “/” represent non-linear associations according to the log-likelihood test of linearity that visually appear to be linear or do not exhibit a specific non-linear pattern. Cells marked with “*” represent positive associations between the exposures and specific cancer types among never smokers. For examples, please refer to Figures 2, 3, 4, S2 and S7.
Abbreviations: BMI: Body mass index; CNS: central nervous system; IARC: International Agency for Research on Cancer; M: Malignant.

Table S4. Diagnostic codes for the definition of cancer cases

Outcome	ICD-10 codes[1]	ICD-9 codes[2]
Head and neck	C00-C14	140-149
Esophagus	C15	150
Stomach	C16	151
Colorectal	C18-C21	153, 154
Liver	C22	155
Gallbladder & biliary tract	C23-C24	156
Pancreas	C25	157
Larynx	C32	161
Trachea, bronchus & Lung	C33-C34	162
Bone and articular cartilage	C40-C41	170
Malignant melanoma of skin	C43	172
Connective and soft tissue[3]	C47, C49	171
Breast premenopausal	C50	174, 175
Breast postmenopausal	C50	174, 175
Cervix Uteri	C53	180
Corpus Uteri	C54-C55	179, 182
Ovary	C56	183, 183.0
Penis[3]	C60	187.1-187.4
Prostate	C61	185
Testis	C62	186
Kidney	C64	189.0
Urinary Tract[3]	C65, C66, C68	189.1-189.9
Bladder	C67	188
Brain and CNS[4]	C70-C72, C75.1-C75.3	191, 192, 194.3, 194.4
Thyroid	C73	193
Hodgkin lymphoma	C81	201
Non-Hodgkin Lymphoma	C82-C86, C96	200, 202
Multiple myeloma	C90	203
Leukemia	C91-C95	204-208
Others and non-specific[3]	C17, C26, C30, C31, C37-C39, C4A, C45, C46, C48, C51, C52, C57, C58, C63, C69, C74, C75.0, C75.4-C75.9, C7A, C76, C80, C88, C97	152, 158-160, 163-165, 176, 181, 184, 187.5-187.9, 190, 194.0, 194.1, 194.5-194.9, 195, 199, 209.1-209.3, 273.3, 279.5

Notes: 1) ICD-10 is the classification system used in the Information System for Research in Primary Care (SIDIAP). 2) ICD-9 is the classification system used in the hospital discharge database. 3) These cancers were used to exclude prevalent cancer cases in the definition of the study population and to

censor individuals during follow-up but were not considered outcomes of interest. 4) Include pituitary gland and pineal gland tumors

Abbreviations: CNS: Central Nervous System; ICD-9: International Classification for Diseases, 9th revision; ICD-10: International Classification for Diseases, 10th revision.

Appendix 2. Description of the multiple imputations' methodology for missing data on body mass index and the covariates of interest

We applied multilevel time raster multiple imputations to have body mass index (BMI) assessments for all the study participants from 18 to at least 40 years of age.

Our primary dataset was in long format and contained as many rows per participant as years with available valid BMI values the individual had. To be considered as valid BMIs, the BMI measurements had to be i) comprised between 15kg/m^2 and 60kg/m^2 (ie, extremely low values could be indicative of an underlying disease, and extremely low/high values could be due to data entry errors in medical records); ii) measured at or after 18 years; iii) not measured during pregnancy. If more than one BMI measurement was available per year, we took the nearest value to the mean for that year.

As a first step to applying the multilevel time raster multiple imputations, we set up the time raster. We chose different time-points to impute BMI for each individual: 18, 30, 40, 55, 70, and 118 (maximum age at which a BMI measurement was observed) years of age. Operationally, one row per time point (eg, 30 years) with a missing value for BMI (which was imputed in the second step of this procedure) was added to the primary dataset for each individual. The information on all the other variables (eg, sex, nationality, etc.) was replicated for each of these rows.

We also added 6 columns that represented a B-spline of degree 1 for the 6 time-points of interest. Considering all valid available BMI measurements, we checked if the age of measurement of the real BMI measurement coincided with one of the time points of interest. In such a case, the B-spline variable corresponding to that column received a 1 (eg, if a person had a real BMI measurement at age 30, then that measurement was attributed a weight of 1/1 for the time point of 30 years, and 0 for all the other time points). In all other cases, the spline coefficients for time points were distributed over two adjacent columns, that summed 1 (eg, if a person had a real BMI measurement at age 35, then that measurement was attributed a weight of 0.50/1 for the time point of 30 years, 0.50/1 for the time point of 40 years, and 0 for all the other time points).

As a second step, we set up the imputation models. Firstly, we specified the model (predictive mean matching with 5 imputations) to impute the categorical variables with missing data at baseline (socioeconomic status, smoking status, alcohol intake). The predictor variables were age at baseline, sex, socioeconomic status (MEDEA deprivation index), smoking status, alcohol intake, geographic region of nationality, the Charlson Comorbidity index, diagnosis of different cancer types, and follow-up time. Secondly, we specified the multilevel model to impute BMI. We used a linear mixed-effects model with PAN implementation, with 5 imputations. The cluster variable was each individual. Level 1 (ie, that can vary within clusters) variables were the age at BMI measurement (B-spline represented) and indicator variables of cancer, cardiometabolic conditions (ie, hypertension, type 2 diabetes, and cardiovascular diseases) and bariatric surgery for which the value was 1 if the individual had been diagnosed with the condition/disease or had the procedure before the BMI assessment (for cancer, 1 year prior), 0 if otherwise (as these conditions and procedures can lead to changes in BMI). Level 2 (ie, that vary between clusters) variables were sex, socioeconomic status, smoking status, alcohol intake, geographic region of nationality, the Charlson Comorbidity index, diagnosis of different cancer types, and follow-up time. We did not include age to evade duplication with its B-spline representation.(1) The model, using the same notation as in Woltman et al.,(2) for subject *i* at time *t* was:

$$\text{BMI}_{ti} = \beta_{0i} + \beta_{1i}(\text{ageBMI}_{ti} + \text{icancer}_{ti} + \text{iHTN}_{ti} + \text{iT2DM}_{ti} + \text{iCVD}_{ti} + \text{iBS}_{ti}) + e_{ti},$$
$$\beta_{ki} = \gamma_{k0} + \gamma_{k1}(\text{sex}_i + \text{SES}_i + \text{smoking}_i + \text{alcohol}_i + \text{nationality}_i +$$
$$\text{Charlson}_i + \text{cancer}_i + \text{follow.up}_i) + u_{ki},$$

where $k = \{0,1\}$, $e_{ti} \sim N(0, {\sigma_t}^2)$, $u_{ki} \sim N(0, {\sigma_k}^2)$.

Abbreviations: BMI: Body mass index; BS: Bariatric surgery; CVD: Cardiovascular disease; HTN: Hypertension; T2DM: Type 2 diabetes mellitus; SES: Socioeconomic status.

After imputing the five BMI trajectories for every individual, we used these trajectories to construct longitudinal BMI-derived exposures among the study participants. Then, we investigated the association between the exposures and cancer risk using Cox Proportional Hazards models. The models' estimates were pooled using Rubin's rule (3). To implement the multilevel time raster multiple imputations and the pooling of results we used the library MICE 3.13.0 available for the software R version 4.0.3.

Figure 1 shows the distribution of observed BMI values (in blue) and of those obtained with the multilevel time raster multiple imputations (in red). For the multiply imputed BMI values that were outside our pre-established limits for valid BMIs (ie, <15kg/m^2 and >60kg/m^2). We assigned values <15kg/m^2 to 15kg/m^2 and those >60kg/m^2 to 60kg/m^2 as post-estimation processing.

Figure 1. Distribution of observed (blue) and multiply imputed (red) BMI values

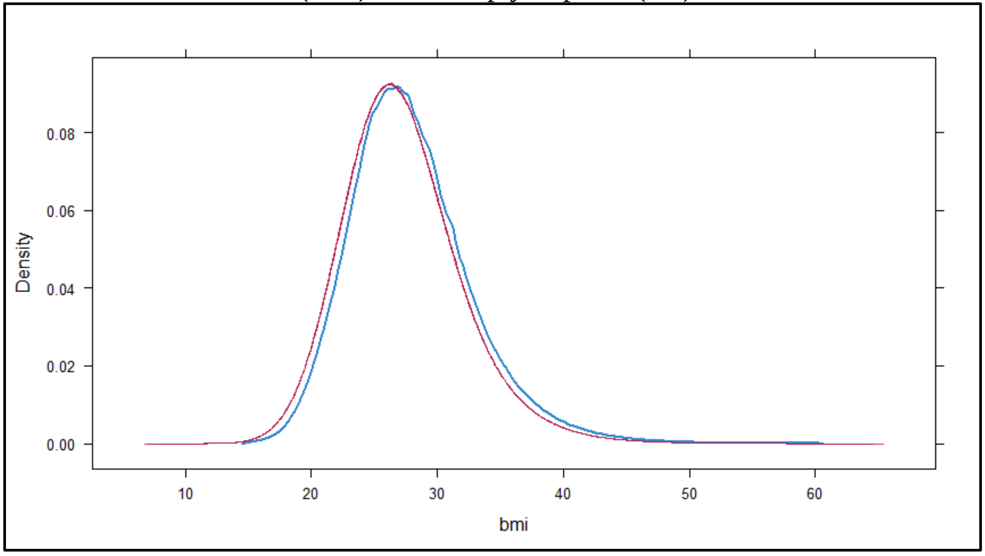

Abbreviations: BMI: body mass index.

Figure 2 shows the results of the multiple imputations for a random sample of 9 individuals. The x-axis corresponds to the time period of interest and the y-axis to the BMI values. We observed real BMI measurements (black dots) throughout follow-up (x-axis) for each individual (box). BMI trajectories are represented with the blue lines (one per imputation) joining the imputed values at each time point (18, 30, 40, 55, 70, and 118) indicated with dotted grey vertical lines, emerging from the x-axis. Vertical colored lines indicate a diagnosis of hypertension, type 2 diabetes mellitus, and/or cardiovascular disease in the corresponding year and of cancer in the year prior.

Figure 2. Five multiply imputed trajectories of BMI for a random sample of 9 individuals (in blue) with observed BMI measurements (black dots).

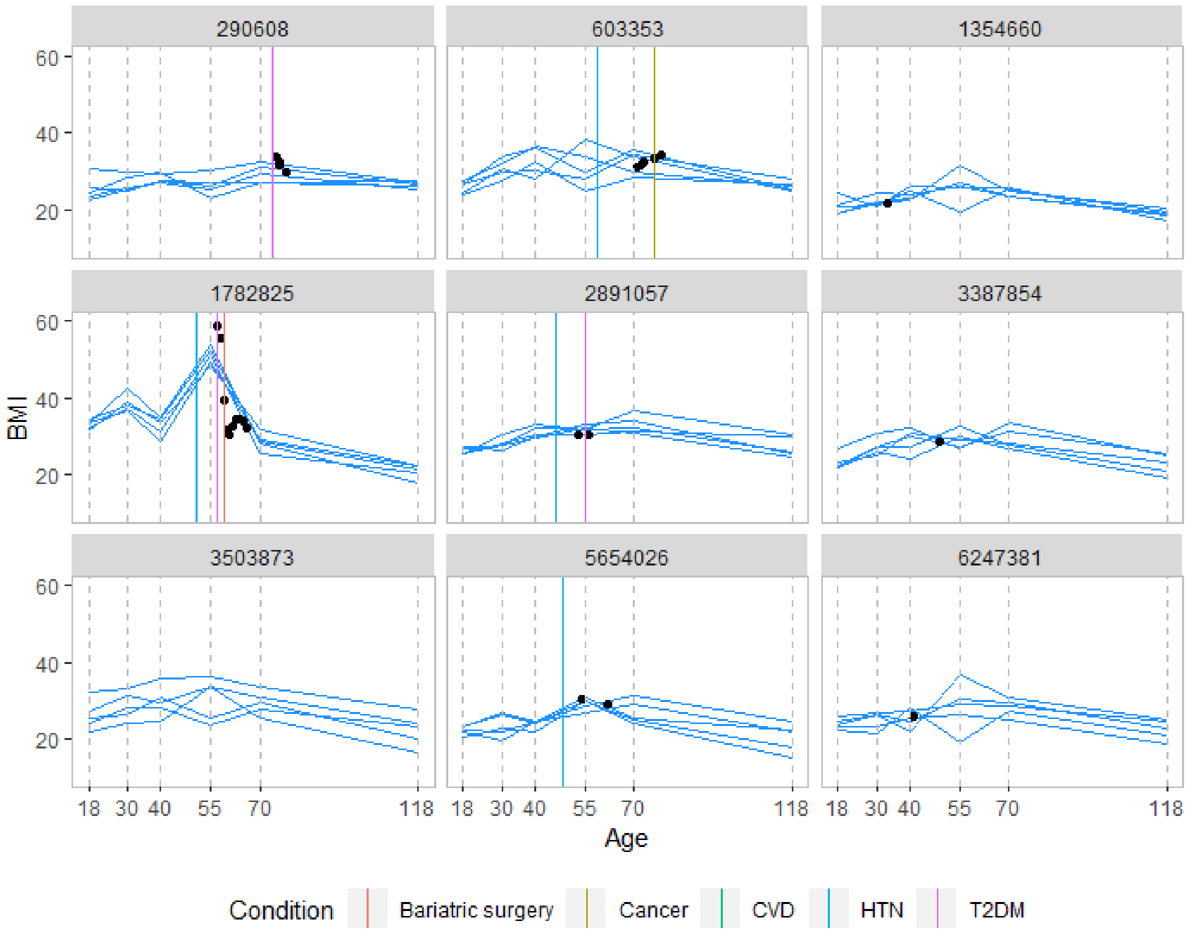

Abbreviations: BMI: body mass index; CVD: Cardiovascular disease; HTN: Hypertension; T2DM: Type 2 diabetes mellitus.

Figure S13. Timeline of this study: illustration with the example of two study participants

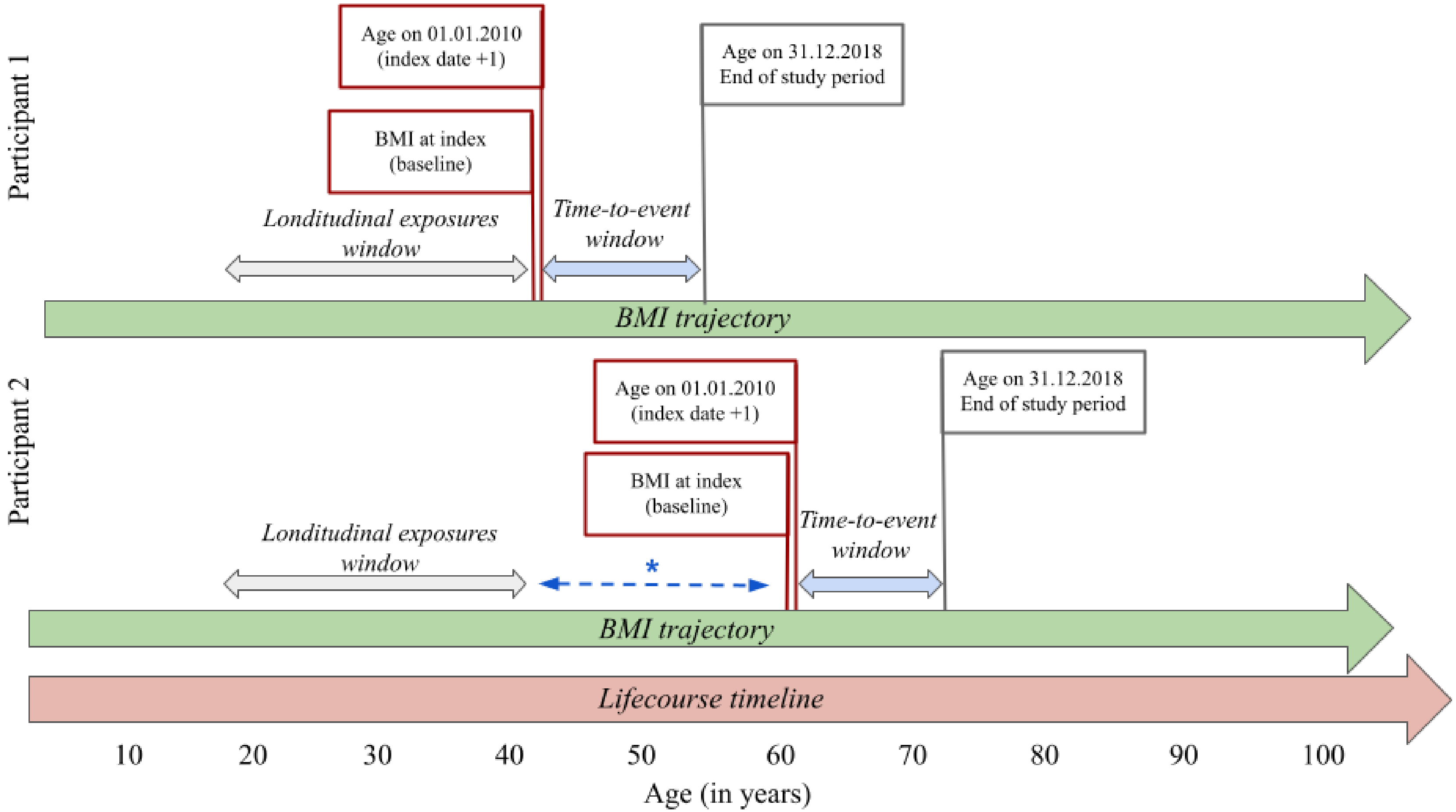

Notes: *We accounted for the difference between the BMI at the end of the longitudinal exposure window and the BMI at the start of the time-to-event window (at index date) in a sensitivity analysis by further adjusting the main models by the difference between the two BMIs. For individuals in the situation of Participant 1 the difference between the two BMIs would be 0, while for someone in the situation of Participant 2, the difference can be ≠ 0. Abbreviations: BMI: body mass index.

Figure S14. Graphical representation for the obtention of longitudinal exposures from BMI trajectories: illustration with the example of an imputed trajectory from a study participant

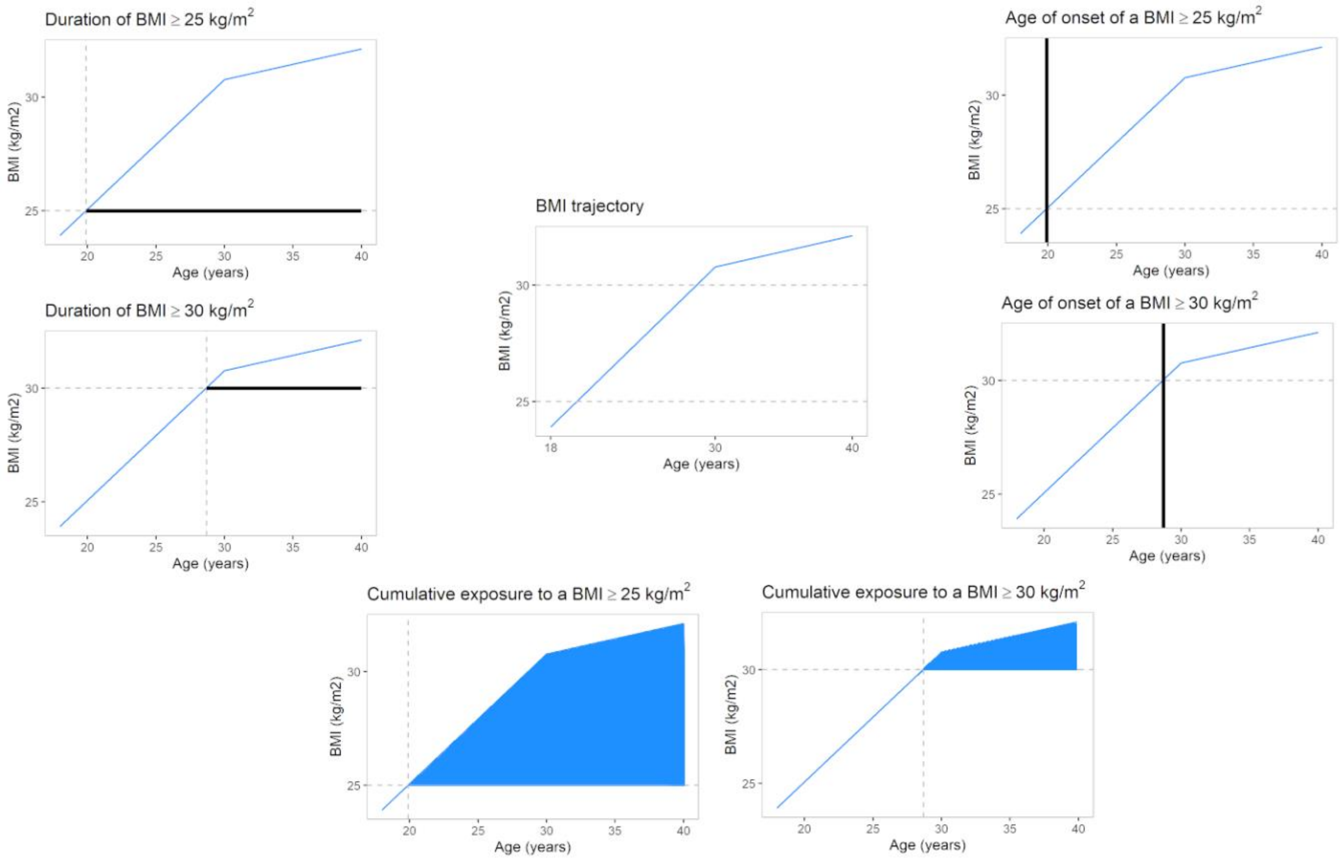

Notes: The duration of BMI≥25 and of ≥30 kg/m^2, respectively, for this individual's trajectory corresponds to the number of years represented by the black bold horizontal line (21 and 12 years, respectively). The cumulative exposure to a BMI ≥25 kg/m^2 (and ≥30) is represented by the blue area under the trajectory (99.3 cumulative overweight-years and 17.3 cumulative obese-years, respectively). The age of onset of BMI≥25 and of ≥30 kg/m^2, respectively, is represented by the black bold vertical line (20 and 29 years of age, respectively).
Abbreviations: BMI: body mass index.

Figure S15. Directed Acyclic Graph for the possible causal effect of body fatness on cancer used to adjust the Cox proportional hazard models

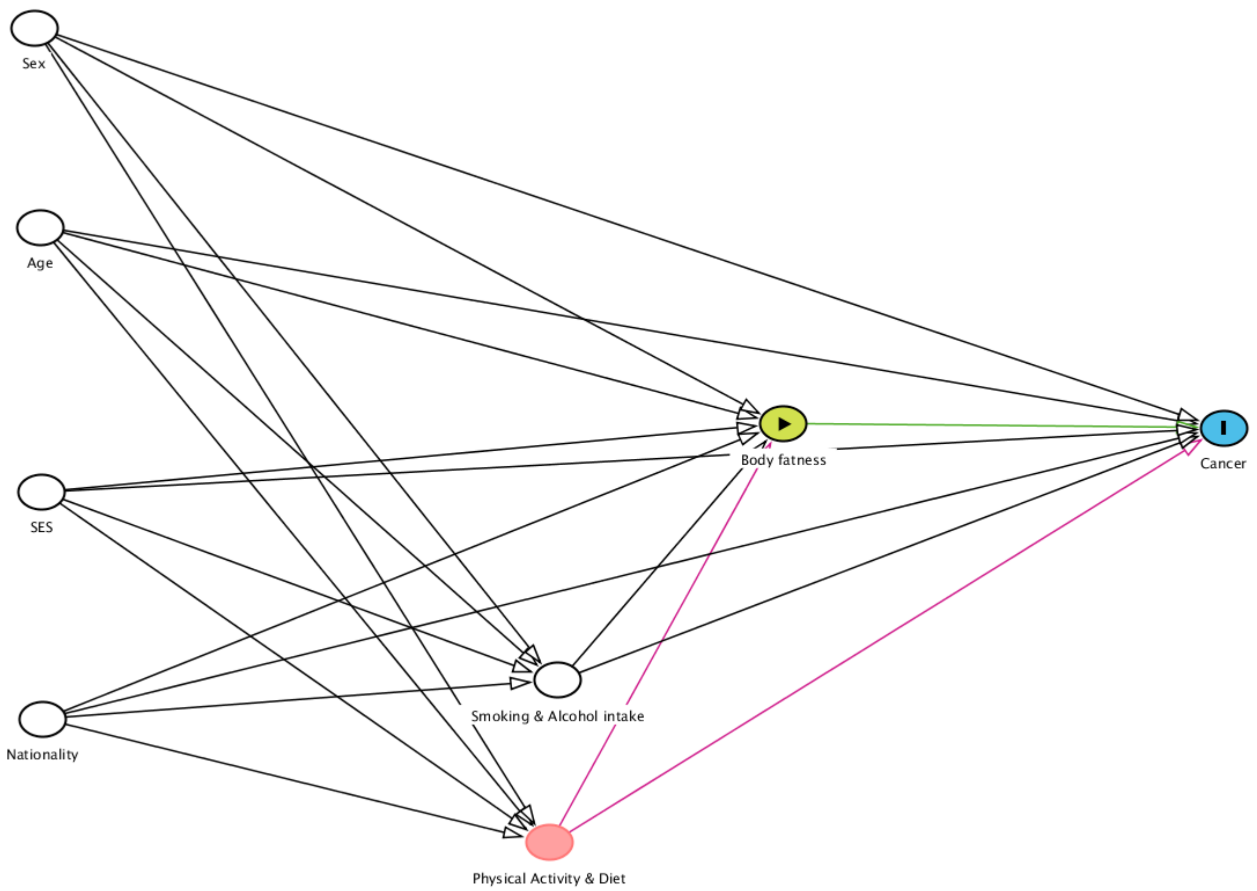

Notes: Green arrows indicate a possible pathway between the exposure and outcome of interest. Red arrows indicate possible confounding pathways in the association between the exposures and outcomes of interest.

Abbreviations: SES: socioeconomic status.

Table S5. N of cases for those who ever lived with a BMI ≥25 and/or ≥30 kg/m^2 throughout the different analyses performed in this study

N corresponding to the following figures:	1, S3, S5, S10A, S11		S4A		S4A		S4B		S4B		S7		S9	S10B		S10C	
**Sub-group of the population:**	**Overall population**		**<65 years**		**≥65 years**		**Male**		**Female**		**Never smokers**		**Overall population**	**≥1 BMI measurement**		**Follow up 3 years after index date**	
**Among those who ever had a BMI:**	**≥25**	**≥30**	**≥25**	**≥30**	**≥25**	**≥30**	**≥25**	**≥30**	**≥25**	**≥30**	**≥25**	**≥30**	**≥25<30**	**≥25**	**≥30**	**≥25**	**≥30**
**Corpus Uteri**	4,463	2,738	2,604	1,587	1,859	1,150	0	0	4,463	2,738	3,756	2,335	3,406	4,023	2,526	3,406	2,108
**Kidney**	5,298	2,435	2,993	1,374	2,306	1,061	3,394	1,453	1,904	981	3,208	1,502	4,773	4,739	2,167	4,190	1,926
**Gallbladder & biliary tract**	1,365	659	423	194	942	464	632	265	733	394	967	484	1,211	1,172	572	1,045	506
**Thyroid**	1,708	792	1,331	621	377	172	442	189	1,266	604	1,196	569	1,514	1,506	695	1,308	606
**Breast postmenopausal**	20,171	9,772	12,094	5,583	8,078	4,189	0	0	20,171	9,772	16,134	8,023	17,721	17,470	8,474	15,446	7,477
**Leukemia**	5,174	2,372	1,981	906	3,193	1,466	2,722	1,149	2,452	1,223	3,614	1,687	4,650	4,501	2,057	3,935	1,808
**Multiple myeloma**	2,562	1,221	919	429	1,643	792	1,248	545	1,314	676	1,856	902	2,289	2,255	1,068	1,996	944
**Testis**	268	108	224	92	45	17	268	108	0	0	138	53	250	225	88	194	75
**Brain and CNS**	2,822	1,284	1,587	701	1,235	583	1,499	624	1,322	660	1,853	868	2,560	2,305	1,040	2,127	955
**Colorectal**	27,621	12,423	12,578	5,609	15,043	6,814	16,333	6,846	11,288	5,577	17,919	8,276	25,131	24,359	10,866	21,408	9,627
**Hodgkin lymphoma**	373	172	250	113	124	59	221	96	153	75	197	92	334	314	145	275	128
**Liver**	4,800	2,112	2,212	964	2,588	1,148	3,203	1,336	1,598	776	2,622	1,190	4,352	4,053	1,770	3,667	1,615
**Ovary**	2,152	1,049	1,299	611	853	437	0	0	2,152	1,049	1,695	840	1,879	1,781	866	1,604	768
**Non-Hodgkin Lymph.**	5,036	2,266	2,625	1,144	2,411	1,123	2,514	1,026	2,521	1,240	3,347	1,560	4,555	4,386	1,957	3,928	1,771
**Malignant melanoma of skin**	4,946	2,197	2,791	1,222	2,154	974	2,459	1,015	2,486	1,181	3,489	1,577	4,520	4,377	1,924	3,807	1,683
**Cervix Uteri**	1,148	552	769	358	379	194	0	0	1,148	552	786	398	1,005	941	455	785	379
**Prostate**	21,351	8,440	9,856	3,911	11,495	4,528	21,351	8,440	0	0	12,333	4,757	20,186	19,560	7,579	15,626	6,150
**Bladder**	15,351	6,479	6,772	2,901	8,579	3,578	12,868	5,295	2,483	1,184	6,911	3,020	14,344	13,783	5,738	11,615	4,899

**Bone and articular cartilage**	559	254	338	151	220	103	274	110	285	144	361	176	513	469	211	435	197
**Pancreas**	4,636	2,036	1,886	805	2,750	1,230	2,298	917	2,337	1,118	3,024	1,363	4,200	3,933	1,696	3,712	1,619
**Breast premenopausal**	1,805	714	1,805	714	0	0	0	0	1,805	714	1,020	424	1,659	1,241	452	1,064	415
**Stomach**	4,353	1,909	1,673	719	2,680	1,190	2,505	1,004	1,848	905	2,848	1,299	3,968	3,746	1,615	3,344	1,477
**Head and Neck**	3,975	1,741	2,212	921	1,763	820	2,544	1,025	1,431	717	2,008	951	3,640	3,371	1,460	2,943	1,288
**Trachea, bronchus & Lung**	15,338	6,229	8,197	3,333	7,141	2,896	12,141	4,780	3,197	1,449	4,963	2,173	14,276	12,515	4,894	11,964	4,828
**Esophagus**	1,343	562	765	322	578	240	1,112	450	231	112	570	247	1,230	1,060	426	1,065	445
**Larynx**	2,312	939	1,526	627	786	311	2,111	845	201	94	591	255	2,164	1,958	776	1,697	678

Notes: In the forest plots shown in this study (Figures 1, S3, S4A, S4B, S5, S7, S9, S10A, S10B, S10C) we display the number of cancer cases among the overall population. However, for the models including the exposures of age of onset of a BMI ≥25 and ≥30 kg/m^2, the individuals included in those analyses are those who ever had a BMI ≥25 and ≥30 kg/m^2, respectively, thus the N of cancers is different from that of the overall population and corresponds to those of this table. Cancer types are ordered by descending ranking of the HRs for BMI at index date. Brain and CNS includes pituitary gland and pineal gland tumors. Abbreviations: BMI: Body Mass Index; CNS: central nervous system; Cum: Cumulative; Lymph: lymphoma.

Table S6. Baseline characteristics of the study population, overall and by having ever had a body mass index ≥25 or ≥30 kg/m², after multiple imputations

	Overall N (%)	Never overweight (BMI<25 kg/m²) N (%)[1]	Ever overweight (BMI≥25 kg/m²) N (%)[1]	Ever obese (BMI≥30 kg/m²) N (%)[1]
	2,645,885	812,369 (30.7)	1,833,516 (69.3)	801,612 (30.3)
**Follow-up time in years, median (IQR)**	9.0 (7.7, 9.0)	9.0 (7.9, 9.0)	9.0 (7.7, 9.0)	9.0 (7.5, 9.0)
**Duration of BMI ≥25 kg/m² in years, median (IQR)[2]**	12.0 (0.0, 23.0)	0.0 (0.0, 0.0)	20.0 (10.0, 23.0)	23.0 (23.0, 23.0)
**Duration of BMI ≥30 kg/m² in years, median (IQR)[2]**	0.0 (0.0, 4.0)	0.0 (0.0, 0.0)	0.0 (0.0, 9.0)	11.0 (5.0, 20.0)
**Cumulative exposure to BMI ≥25 kg/m² in cumulative overweight-years, median (IQR)[2,3]**	16.4 (0.0, 73.7)	0.0 (0.0, 0.0)	45.9 (13.7, 103.3)	113.5 (78.3, 163.3)
**Cumulative exposure to BMI ≥30 kg/m² in cumulative obese-years, median (IQR)[2,3]**	0.0 (0.0, 2.2)	0.0 (0.0, 0.0)	0.0 (0.0, 12.4)	17.4 (4.2, 51.9)
**Age of onset of BMI ≥25 kg/m² in years, median (IQR)[2,4]**	20.0 (18.0, 29.0)	-	20.0 (18.0, 29.0)	18.0 (18.0, 18.0)
**Age of onset of BMI ≥30 kg/m² in years, median (IQR)[2,4]**	29.0 (21.0, 35.0)	-	29.0 (21.0, 35.0)	29.0 (21.0, 35.0)
**BMI at index date in kg/m², median (IQR)[2,5]**	27.6 (24.2, 31.1)	23.0 (20.7, 24.9)	29.4 (26.8, 32.5)	32.5 (30.5, 35.2)
**Age in years, median (IQR)**	56.0 (47.0, 68.0)	55.0 (46.0, 66.0)	57.0 (47.0, 70.0)	58.0 (48.0, 71.0)
**Male sex, n (%)**	1,241,523 (46.9)	362,147 (44.6)	879,376 (48.0)	358,172 (44.7)
**Nationality**				
Spanish	2,495,536 (94.3)	766,176 (94.3)	1,729,360 (94.3)	756,163 (94.3)
Global North	51,320 (1.9)	17,049 (2.1)	34,271 (1.9)	14,834 (1.9)
Global South	99,029 (3.7)	29,145 (3.6)	69,884 (3.8)	30,616 (3.8)
**MEDEA deprivation index, n (%)[2]**				
Quintile 1 (least deprived)	472,049 (17.8)	170,403 (21.0)	301,646 (16.5)	120,028 (15.0)
Quintile 2	429,823 (16.2)	136,784 (16.8)	293,039 (16.0)	124,672 (15.6)
Quintile 3	416,465 (15.7)	123,903 (15.3)	292,562 (16.0)	128,865 (16.1)
Quintile 4	401,681 (15.2)	112,463 (13.8)	289,218 (15.8)	131,747 (16.4)
Quintile 5 (most deprived)	361,665 (13.7)	96,963 (11.9)	264,702 (14.4)	125,063 (15.6)
Rural	564,201 (21.3)	171,853 (21.2)	392,348 (21.4)	171,237 (21.4)
**Smoking status, n (%)[2]**				
Never smoker	1,663,154 (62.9)	486,100 (59.8)	1,177,054 (64.2)	529,478 (66.1)

Former smoker	390,711 (14.8)	110,853 (13.6)	279,858 (15.3)	122,089 (15.2)
Current smoker	592,020 (22.4)	215,416 (26.5)	376,604 (20.5)	150,046 (18.7)
**Alcohol intake, n (%)[2]**				
No risk	1,663,281 (62.9)	501,729 (61.8)	1,161,553 (63.4)	526,049 (65.6)
Low risk	894,238 (33.8)	283,422 (34.9)	610,816 (33.3)	249,140 (31.1)
High risk	88,366 (3.3)	27,218 (3.4)	61,147 (3.3)	26,423 (3.3)
**Charlson comorbidity index, n (%)**				
0	1,250,781 (47.3)	439,775 (54.1)	811,006 (44.2)	323,454 (40.4)
1	892,103 (33.7)	243,404 (30.0)	648,699 (35.4)	302,468 (37.7)
2	357,217 (13.5)	94,760 (11.7)	262,457 (14.3)	121,705 (15.2)
≥ 3	145,784 (5.5)	34,430 (4.2)	111,354 (6.1)	53,986 (6.7)
**Cause of exit from the study, n (%)**				
End of study	1,865,496 (70.5)	577,856 (71.1)	1,287,640 (70.2)	557,511 (69.5)
Transferred out of the SIDIAP	291,641 (11.0)	94,850 (11.7)	196,791 (10.7)	85,001 (10.6)
Death	250,914 (9.5)	71,727 (8.8)	179,187 (9.8)	83,541 (10.4)
Any cancer[6]	237,834 (9.0)	67,935 (8.4)	169,899 (9.3)	75,559 (9.4)
**Cancer outcomes, n (%)**	225,396 (8.5)	64,466 (7.9)	160,930 (8.8)	71,456 (8.9)

Notes: 1) This categorization was done in the 5 datasets obtained after performing the multiple imputations. For visualization purposes and in order for the categorical variables to add up to 2,645,885 we divided the n for the categorical variables by 5. 2) The exposures of interest, the MEDEA deprivation index, smoking status, and alcohol intake were calculated using the multiple imputation approach, with 5 data sets created. For visualization purposes, we divided the n for the categorical variables by 5. 3) This indicator was calculated by adding the difference between the BMI measurements that were ≥25 (≥30, for obesity) kg/m^2 and 24.9 (29.9) kg/m^2 for every year lived with a BMI ≥25 and ≥30, respectively. 4) Age of onset of a BMI ≥25 (and ≥30) kg/m^2 is only available for individuals who ever had a BMI ≥25 (≥30) kg/m^2. 5) BMI assessment at the start of the time-to-event analysis (baseline BMI). 6) Any cancer does not include non-melanoma skin cancer.

Abbreviations: BMI: Body Mass Index; IQR: Interquartile range; MEDEA: Mortalidad en áreas pequeñas Españolas y Desigualdades Socioeconómicas y Ambientales; SIDIAP: Information System for Research in Primary Care.

Appendix 3. STROBE Statement checklist

	Item No	Recommendation	Page No
**Title and abstract**	1	(*a*) Indicate the study's design with a commonly used term in the title or the abstract	1
		(*b*) Provide in the abstract an informative and balanced summary of what was done and what was found	2
**Introduction**			
Background/rationale	2	Explain the scientific background and rationale for the investigation being reported	3-4
Objectives	3	State specific objectives, including any prespecified hypotheses	4
**Methods**			
Study design	4	Present key elements of study design early in the paper	11
Setting	5	Describe the setting, locations, and relevant dates, including periods of recruitment, exposure, follow-up, and data collection	11
Participants	6	(*a*) Give the eligibility criteria, and the sources and methods of selection of participants. Describe methods of follow-up	11
		(*b*) For matched studies, give matching criteria and number of exposed and unexposed	-
Variables	7	Clearly define all outcomes, exposures, predictors, potential confounders, and effect modifiers. Give diagnostic criteria, if applicable	11-14
Data sources/ measurement	8*	For each variable of interest, give sources of data and details of methods of assessment (measurement). Describe comparability of assessment methods if there is more than one group	11-14
Bias	9	Describe any efforts to address potential sources of bias	15
Study size	10	Explain how the study size was arrived at	-
Quantitative variables	11	Explain how quantitative variables were handled in the analyses. If applicable, describe which groupings were chosen and why	14-15
Statistical methods	12	(*a*) Describe all statistical methods, including those used to control for confounding	14-15
		(*b*) Describe any methods used to examine subgroups and interactions	15
		(*c*) Explain how missing data were addressed	14-15
		(*d*) If applicable, explain how loss to follow-up was addressed	11
		(*e*) Describe any sensitivity analyses	15
**Results**			
Participants	13*	(a) Report numbers of individuals at each stage of study—eg numbers potentially eligible, examined for eligibility, confirmed eligible, included in the study, completing follow-up, and analysed	4
		(b) Give reasons for non-participation at each stage	4
		(c) Consider use of a flow diagram	Supp.
Descriptive data	14*	(a) Give characteristics of study participants (eg demographic, clinical, social) and information on exposures and potential confounders	4
		(b) Indicate number of participants with missing data for each variable of interest	Table 1.
		(c) Summarise follow-up time (eg, average and total amount)	4 Table 1.
Outcome data	15*	Report numbers of outcome events or summary measures over time	Table 1.
Main results	16	(*a*) Give unadjusted estimates and, if applicable, confounder-adjusted estimates and their precision (eg, 95% confidence interval). Make	5-6 Supp.

		clear which confounders were adjusted for and why they were included	
		(*b*) Report category boundaries when continuous variables were categorized	5-6
		(*c*) If relevant, consider translating estimates of relative risk into absolute risk for a meaningful time period	-
Other analyses	17	Report other analyses done—eg analyses of subgroups and interactions, and sensitivity analyses	Supp.
**Discussion**			
Key results	18	Summarise key results with reference to study objectives	6-7
Limitations	19	Discuss limitations of the study, taking into account sources of potential bias or imprecision. Discuss both direction and magnitude of any potential bias	9-10
Interpretation	20	Give a cautious overall interpretation of results considering objectives, limitations, multiplicity of analyses, results from similar studies, and other relevant evidence	6-10
Generalisability	21	Discuss the generalisability (external validity) of the study results	9-10
**Other information**			
Funding	22	Give the source of funding and the role of the funders for the present study and, if applicable, for the original study on which the present article is based	20

Supplementary references

1. Buuren S van. Flexible Imputation of Missing Data. Second Edition [Internet]. Stef van Buuren. 2018 [cited 2021 Nov 12]. Available from: https://stefvanbuuren.name/publication/2018-01-01_vanbuuuren2018/
2. Woltman, H., Feldstain, A., MacKay, J. C., & Rocchi, M. (2012). An Introduction to Hierarchical Linear Modeling. Tutorials in Quantitative Methods for Psychology, 8, 52-69.. Quantitative Methods Psychology tutorial. 8. 52-69.
3. Rubin, D.B. *Multiple Imputation for Nonresponse in Surveys*. J. Wiley & Sons, New York; 1987.
